# Identification of the MicroRNA Repertoire in TLR-Ligand Challenged Bubaline PBMCs as a Model of Bacterial and Viral Infection

**DOI:** 10.1371/journal.pone.0156598

**Published:** 2016-06-03

**Authors:** Jasdeep Singh, C. S. Mukhopadhyay, Simarjeet Kaur, Puneet Malhotra, R. S. Sethi, R. K. Choudhary

**Affiliations:** 1 School of Animal Biotechnology, Post Graduate Institute of Veterinary Education and Research, Guru Angad Dev Veterinary and Animal Sciences University, Ludhiana, Punjab, India; 2 Department of Animal Genetics and Breeding, Guru Angad Dev Veterinary and Animal Sciences University, Ludhiana, Punjab, India; Huazhong University of Science and Technology, CHINA

## Abstract

In the present study, we used high-throughput sequencing, miRNA-seq, to discover and explore the expression profiles of known and novel miRNAs in TLR ligand-stimulated vis-à-vis non-stimulated (i.e. Control) peripheral blood mononuclear cells (PBMCs) isolated from blood of healthy Murrah buffaloes. Six small RNA (sRNA) libraries were multiplexed in Ion Torrent PI chip and sequenced on Ion Proton System. The reads obtained were aligned to the *Bos taurus* genome (UMD3.1 assembly), which is phylogenetically closest species to buffalo (*Bubalus bubalis)*. A total of 160 bovine miRNAs were biocomputationally identified in buffalo PBMCs and 130 putatively novel miRNAs (not enlisted in the bovine mirBase) were identified. All of these 290 miRNAs identified across the six treatment and control samples represent the repertoire of novel miRNAs for the buffalo species. The expression profiles of these miRNAs across the samples have been represented by sample dendrogram and heatmap plots. The uniquely expressed miRNAs in each treatment and control groups were identified. A few miRNAs were expressed at very high levels while the majority of them were moderately expressed. The miRNAs bta-miR-103 and -191 were found to be highly abundant and expressed in all the samples. Other abundantly expressed miRNAs include bta-miR-19b, -29b, -15a, -19a, -30d, -30b-5p and members of let family (let 7a-5p, let 7g & let 7f) in LPS and CpG treated PBMCS and bta-miR-191, -103 & -19b in Poly I:C stimulated PBMCs. Only one novel miRNA (bta-miR-11039) out of 130 identified putatively novel miRNAs, was expressed in all the six samples and differentially expressed (>2- fold) miRNAs were identified. Six of the differentially expressed miRNAs across the groups (bta-miR-421, bta-let-7i, bta-miR-138, bta-miR-21-5p, bta-miR-222 and bta-miR-27b) were subsequently confirmed by TaqMan quantitative reverse transcription polymerase chain reaction (qRT-PCR). Furthermore, the target genes of differentially expressed miRNAs were enriched for the roles in innate immunity and TLR signaling pathways. This maiden study on profiling and cataloguing of bubaline miRNAs expressed in TLR-ligand stimulated PBMCs will provide an important reference point for future studies on regulatory roles of miRNAs in immune system of buffaloes.

## Introduction

MicroRNAs (miRNAs) are small (∼22 bases), non-coding, endogenous, single-stranded RNAs that are expressed in plants, animals as well as in algae and certain viruses [1–2]. MicroRNAs have been implicated as key regulators of immune system, development and physiological functions [3]. Increasing evidence suggests that miRNA expression plays an important role in host–pathogen interactions through modulation of both innate and acquired immune responses [4–5]. The innate immune system is activated following recognition of pathogen-associated molecular patterns (PAMPs) by host pattern recognition receptors, such as toll-like receptors (TLRs) which recognize both extracellular and intracellular PAMPs [6]. The TLR-signaling pathways are regulated by the expression of variety of miRNAs. For example, Lipopolysaccharide (LPS) treatment results in decrease of let-7 expression along with increased TLR4 expression [7]. Moreover, miR-105 and miR-19 are predicted to target mRNA encoding TLR2 leading to its decreased expression and further decrease in cytokine production in primary human keratinocytes [8].

The majority of miRNAs target the transcripts for intermediate molecules involved in TLR signaling instead of directly targeting the TLR-transcripts [9–12]. For example, miR-146 targets IL-1R-associated kinase1 (IRAK1), IRAK2 and TNFR-associated factor 6 (TRAF6) that activate NF-κB production following TLR signaling cascade [13–14]. Moreover, the myeloid differentiation primary-response protein 88 (MYD88), TAK1-binding protein 2 (TAB2), and TRAF6 are the direct targets of miR-155 [15]. Furthermore, miR-9 directly targets mRNA of the NF-kB subunit important for NF-kB assembly [12].

Considering the role of miRNAs in innate immune response, the present study was designed to discover and explore the miRNA profile of buffalo PBMCs in response to ligands for the bubaline toll like receptors viz. TLR4 ligand (liposaccharides (LPS)), TLR 9 ligand (CpG ODN) to mimic bacterial infection and TLR3 ligand (poly I:C) for simulating viral infection *in vitro*. Until the time of reporting of this study there has been no published miRNA dataset available for bubaline species, so this analysis of miRNA expression in PBMCs challenged with bacterial and viral ligands will contribute significantly to the current knowledge of miRNAs playing roles in disease pathogenesis.

## Materials and Methods

### Blood Samples

All the blood samples, for the current study, were aseptically collected in 50 ml centrifuge tube, containing 500 μl of 0.5M EDTA and the experiments were performed under the guidelines of Institutional Animal Ethics Committee (IAEC), Guru Angad Dev Veterinary and Animal Sciences University (GADVASU) (VMC/12/3901-35; Dated 6^th^ August’ 2012). The work stated in the manuscript was certified by the IAEC of GADVASU, Ludhiana, India for the SERB-DST sponsored project. The peripheral blood collection was done strictly according to the recommendations of the IAEC of our Institution. Peripheral blood (about 15 ml each) was drawn from the jugular vein of healthy female Murrah buffaloes (n = 4 per group; total six groups i.e. 3 treatment and 3 respective control groups), maintained at the Dairy farm, GADVASU. Blood samples were immediately brought to the laboratory in ice and were processed for PBMCs isolation.

### PBMCs culture and stimulation with TLR ligands

PBMCs were isolated from the blood samples of healthy animals, pooled for each group and cultured in 30ml of RPMI growth media (including 10%FBS, antibiotic mix) in 75cm^2^ culture flask. In an another experiment, the PBMCs were challenged separately with TLR ligands (LPS, and Poly I:C) in combination of different doses and incubation time and the expression of their respective receptor genes (TLR4 and TLR3) was studied using real time PCR. Cultured PBMCs (isolated from blood of healthy animals; n = 4 and pooled) were stimulated with two different doses of poly I:C (Final conc. 10μg/ml and 50μg/ml) for different time intervals: 1hr, 3hr, 6hr, 12hr, 18hr and 24 hr. Like-wise four different LPS doses (10ng/ml, 100ng/ml, 1000ng/ml and 2000ng/ml) were used to stimulate the PBMCs cultures (isolated from blood of healthy animals; n = 4 and pooled). Normal, untreated PBMCs just at start of incubation time (0 hr) and at end of each interval time was taken as control [16]. ODN 2007 is a class B CpG ODN with a preference for bovine and porcine TLR9. CpG ODN dosage were chosen as recommended by suppliers (Invivogen) and previous studies. CpG ODN (10μg/ml) stimulation results in increased lymphocyte proliferation within the bovine peripheral blood mononuclear cells (PBMCs) [17–19].

For the present experiment, the cultured PBMCs were given treatment and incubated for specified time (Table 1) at which optimum/maximum expression of their respective TLR gene was observed in standardization experiment. After the specified incubation period, the PBMCs were harvested and processed further for extraction of miRNA enriched small RNA (sRNA).

**Table 1:** Standardized dose of TLR ligands and incubation period used for stimulating the cultured PBMCs.

S.N.	Treatment	Control
1.	TLR4 Ligand (LPS)@ 100ng/ml for 6hr	No stimulation, 6hr
2.	TLR3 Ligand (Poly I:C) @50μg/ml for 12hr	No stimulation, 12hr
3.	TLR9 Ligand (CpG ODN 2007) @ 10μg/ml for 12hr	No stimulation, 12hr

### sRNA isolation and library preparation

The RNA fraction enriched for sRNAs (including miRNAs) was purified from the pelleted PBMCs using the *mir*Vana miRNA Isolation Kit (Life Technologies, USA). The sRNA samples were outsourced to Bioserve Biotechnologies (A CGI company, Hyderabad, India), for next generation sequencing. Initially, the quality and quantity of the purified sRNAs were assessed using Agilent 2100 Bioanalyzer using sRNA LabChip kits (Agilent Technologies, USA). The concentrations of sRNA and miRNA ranged between ~18,334–105,216 pg/μl and ~975–8666pg/μl, respectively (S1 Table). Representative cDNA libraries were constructed using Ion Total RNA-Seq Kit v2 (Life Technologies). The yield and size distribution of the amplified DNA was assessed on an Agilent2100 Bioanalyzer instrument with the Agilent DNA 1000 Kit. Each of the barcoded cDNA libraries were diluted to the same molar concentration (nM) and all the six libraries were sequenced by multiplexing using Ion-Torrent PI chip on the Ion Proton System, based on semiconductor high-throughput sequencing.

### Analysis of RNA-seq data

#### Pre-processing of the reads

The initial raw data was pre-processed using FASTX tool kit (http://hannonlab.cshl.edu/fastx_toolkit/), namely, FASTX-clipper tool (for trimming the adapter sequence) and FASTX- quality trimmer (to remove the low quality bases). The reads which were of length less than16 bases, more than 35 bases and reads matching tRNA and rRNA were discarded. The quality value for all the processes is taken as -Q 33.

#### Alignment to reference genome and identification of known and novel miRNAs

Reads which passed all quality control steps were aligned to the bovine genome (UMD3.1) [20]. UEA SRNA workbench (http://srna-workbench.cmp.uea.ac.uk/) was used to identify the known and novel miRNAs [21]. The identifications of mature miRNA and the target transcripts for the query sequences were identified using mirBase microRNA database (http://www.mirbase.org/). As the reads have been aligned to the bovine genome assembly, the miRNAs thus identified as known and novel are in terms of bta-miRNA sequences, while all the miRNAs being reported here are novel for buffalo.

#### Identification of common and unique miRNAs

The Venn diagrams representing the common and unique miRNAs among all the six groups combined and between the treatment and the respective control samples were constructed using online web-tool (http://www.interactivenn.net/) and using R Program (v. 3.2.0) package Venn Diagram v. 1.6.9, respectively.

#### Expression analysis

To compare differentially expressed bubaline miRNAs between TLR ligand stimulated- and control-PBMC cells, the read counts of each identified miRNAs was normalized to the total number of miRNA reads and transformed to logarithmic scale. R program (Version 3.0.2) was used to create the box-plots showing the abundance of the identified known and novel miRNAs across the samples. Expression heatmaps (on colour scale bar; white-orange-red; representing low-medium-high expression, respectively) of the miRNAs for the experimental samples was constructed using WGCNA package [22] of R program (v. 3.0.2). The sample groups as well as miRNAs were clustered together based on similar expression levels and represented by colored sample dendrogram.

#### Quantification of miRNA expression

Expression of six miRNAs was quantified by real time PCR using TaqMan microRNA assays. For each treatment versus control group; 15 ml blood was again collected from unrelated, healthy animals (biological replicates, n = 3), processed for PBMCs isolation separately and cultured in 6-well plates. Cultured PBMCs were stimulated as per the respective dosage and time intervals of the ligands and thereafter processed for total RNA (including miRNAs) individually, using miRVana miRNA isolation kit (Ambion).

#### Reverse Transcription (RT) Reaction

Total RNAs (including miRNAs) were converted into cDNAs using by TaqMan microRNA reverse transcription kit. This kit converts total RNAs into cDNA using specific miRNA primers which are provided with respective Taqman MicroRNA Assays.For each sample, a 15μl reaction was setup on ice having 7 μl of RT master mix (0.25 mM dNTPs, 3.33 U/μL multiscribe RT enzyme, 1X RT buffer, 0.25 U/μL RNAse inhibitor), 3 μl of specific reverse primer and 5 μl of RNA sample. Reaction tubes were incubated at temperature of 16°C for 30 mins, 42°C for 30mins and last at 85°C for 5mins in a Veriti thermal cycler (Applied Biosystems).

#### Real Time PCR Detection

In the next step, 0.8 μL of the product from RT reaction (diluted 1:2) was combined with 0.5 μL of a 20X TaqMan MicroRNA Assay (forward primer, reverse primer, and probe) and 5 μL of 2X TaqMan Universal PCR Master Mix, in a 10-μL final volume. Real-time PCR was performed using BioRAD CFX96-Realtime PCR System with the standard TaqMan microRNA assays protocol i.e. cycling conditions of 95°C for 10 min, followed by a total of 40 cycles of 95°C for 15 sec and 60°C for 60 sec. Each TaqMan Assay was run in triplicate. The relative quantification of mature miRNA expression was normalized to the expression of bovine miR-191 control assays. For each assay, a no-template control (NTC) reaction was also included.

#### Target prediction and functional analysis

The potential target genes of cattle species for the differentially expressed miRNAs were predicted using three tools: TargetScan v6.2 (http://www.targetscan.org/) [23–25], miRDB [26, 27] and PicTar [28]. The target transcripts were screened according to their functions and their roles in innate immunity and TLR signaling were identified. On this basis the miRNAs that may be involved in the regulation of the immune response and disease pathogenesis were also indentified. Gene ontology and functional classification of the miRNA targets was performed using PANTHER Classification System (http://pantherdb.org/) [29].

## Results

The primary aim of this study was to identify and catalogue the bubaline miRNAs from PBMCs challenged with TLR ligands. For this purpose, reads obtained from the NGS run were aligned to the genome of taurine cattle (*Bos taurus*). Hence miRNAs identified as the known and the novel is with respect to the taurine miRNAs (at mirBase), while all the miRNAs identified are novel for the buffalo species. To the extent of our knowledge, this is the first work identifying the buffalo miRNA repertoire in PBMCs challenged with the TLR-ligands. All the miRNAseq data (FASTQ format) was submitted to NCBI under Bioproject number: PRJNA297948 and the data are made fully available without restriction. The individual NCBI-SRA Accession numbers for respective samples are: SRR3144565 (LPS treated 6hr), SRR3362227 (poly I:C treated 12hr), SRR3362628 (CpG treated 12hr), SRR3362629 (LPS control 6hr), SRR3362630 (poly I:C control 12hr) and SRR3382568 (CpG control 12hr).

### Known and novel miRNAs

The total known and novel miRNAs identified in each treatment and control samples have been enlisted in Table 2. Altogether 160 known miRNAs were identified and were assigned names according to their *Bos taurus* miRNA ID’s. Additionally, 130 putative novel miRNAs were identified based on p-values with a predicted mature sequence showing no homology to miRNAs in miRBase. As these 130 miRNA sequences did not have any taurine homologs, these miRNAs were assigned arbitrary numbers.

**Table 2:** The detail of the number of filtered reads and unique vis-à-vis novel miRNAs obtained post sequencing and bioinformatics analysis of the miRNA-Seq data.

Sample	Description	Total initial Reads** (after filtration)	Total miRNA#	UniquemiRNA^$^	NovelmiRNA*	UniqueNovelmiRNA
1	LPS Treatment 6Hr	7795909	155	138	56	41
2	Poly I:C Treatment 12 Hr	18197787	154	134	116	82
3	CpG Treatment 12 Hr	6506097	114	95	39	24
4	LPS Control 6Hr	7968649	112	98	39	25
5	Poly I:C Control 12 Hr	5495294	146	129	57	43
6	CpG Control 12 Hr	3818034	115	102	61	38

** Total Initial Reads (after filtration).

^$^ Unique refers to the miRNAs that are not repeated in the report.

* Novel means the miRNAs sequences which have no homologues in miRBase data on taurine cattle (*Bos taurus*) or any other species.

^#^ The miRNA sequences have been identified taking *Bos taurus* genome (Bta UMD 3.1) as reference sequence.

The detailed list of known and novel miRNAs, including with their ID’s, mature miRNA sequence, respective pre-miRNA sequences and length; position of miRNAs within the hairpin, GC percentage and P values, have been given in the Supplementary information (S2 and S3 Tables, respectively). Additionally, three novel miRNAs (bta-miR-11033, bta-miR-12051 and bta-miR-16059) have been identified whose mature sequence does not match with the mature miRNA sequences in mirBase database but matches were found for their respective pre-miRNA/ hairpin sequences (S4 Table).

### Uniquely expressed miRNAs

**Fig 1:**
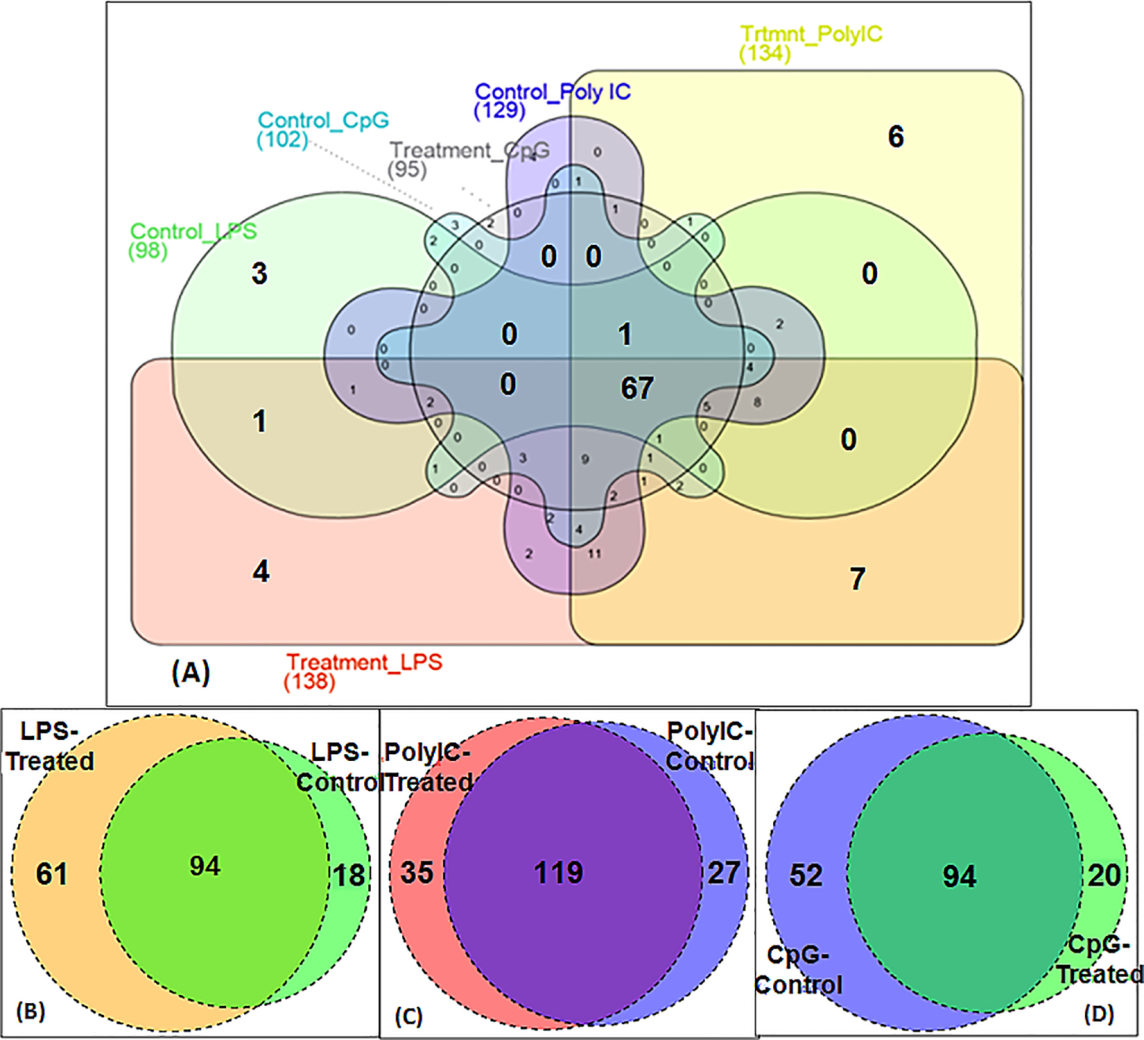
Venn diagrams representing common and unique miRNAs across the groups. All the four treatment and control groups together (A); Comparison of treatment and control groups viz. LPS stimulated and non-stimulated control (B), PolyI:C stimulated and non-stimulated control (C), and CpG stimulated and non-stimulated control (D).

Comparative analysis between three pairs of TLR-ligand groups (treatment and control) revealed the uniquely expressed miRNAs:(a) 61 in LPS-challenged and 18 in un-treated-control samples; (b) 35 in Poly I:C-challenged and 27 in un-treated-control samples; and (c) 52 in CpG-challenged and 20 in un-treated-control samples, respectively (Fig 1). These unique miRNAs could have potential role in conferring tolerance or susceptibility to specific infections.

### Expression Analysis

**Fig 2:**
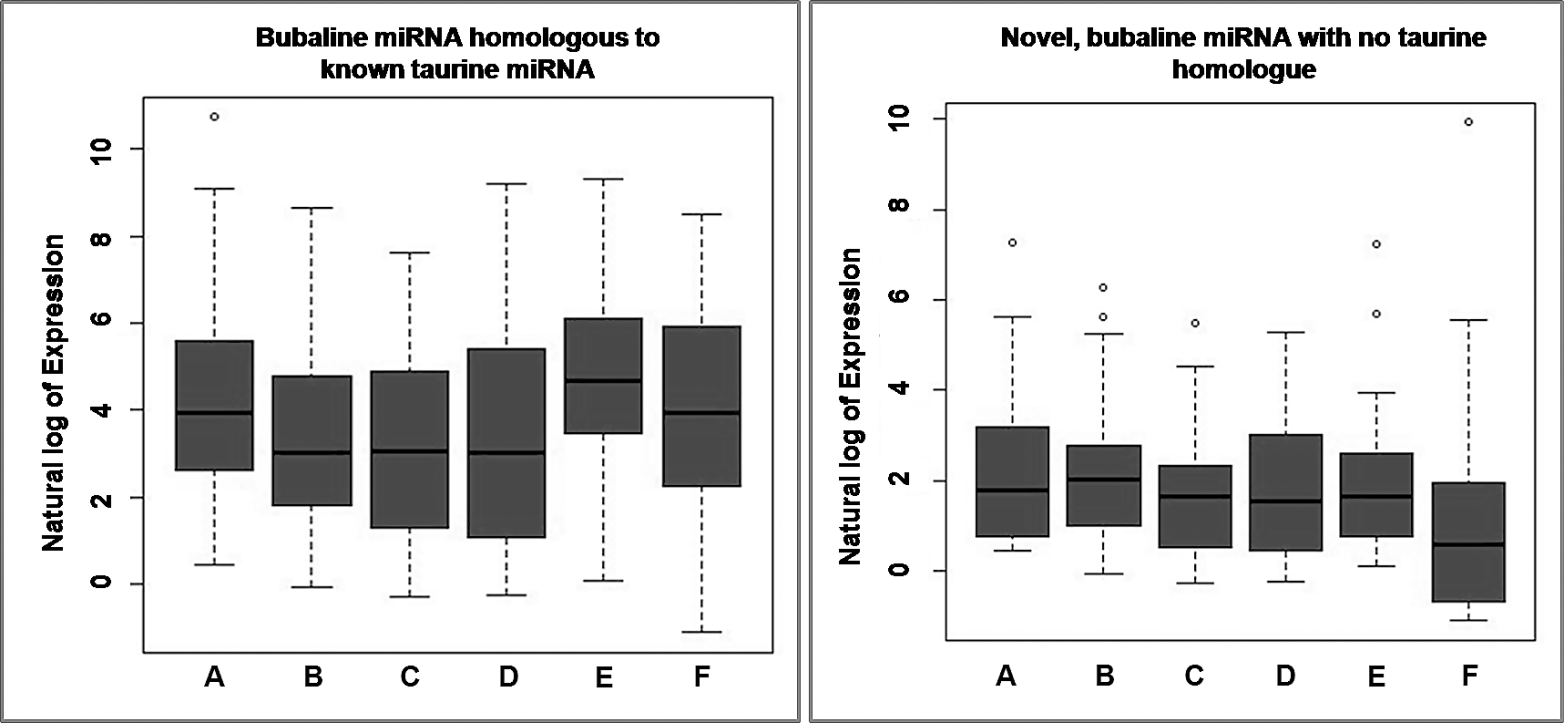
Box-plot showing abundance of known and novel miRNAs across TLR ligand treated and control samples. LPS stimulated PBMCs (A), poly I:C stimulated PBMCs (B), CpG ODN stimulated PBMCs (C), non-LPS-stimulated control PBMCs (D), non-poly I:C-stimulated control PBMCs (E), non-CpG stimulated control PBMCs (F). The central lines inside the boxes indicate median values, box width indicates 25% and 75% quartile ranges around the median, “T” indicates the maximum and minimum values, and the dots represent outliers.

**Fig 3:**
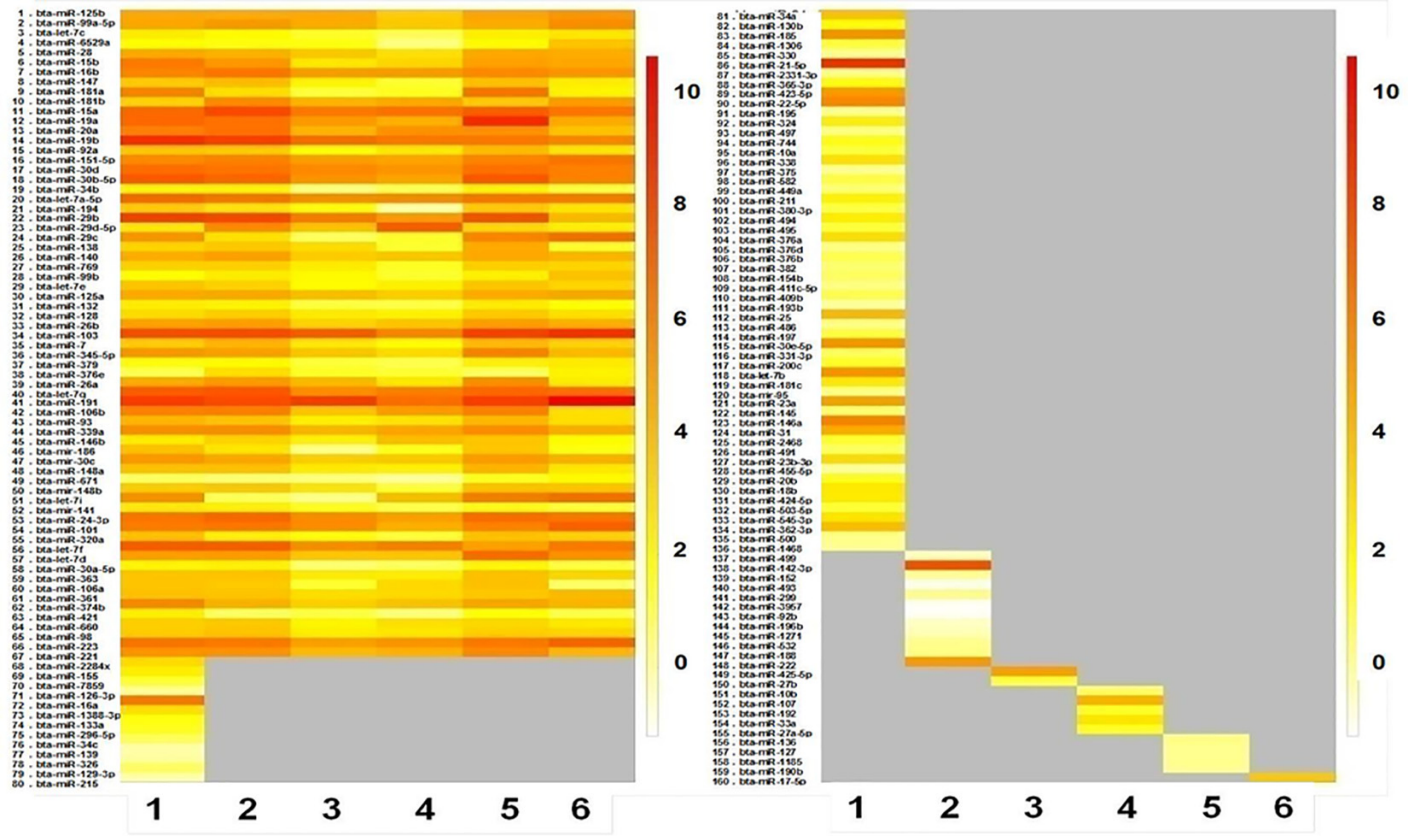
Heatmap of known miRNA expression across the TLR stimulated and control samples. LPS stimulated PBMCs (A), non-LPS-stimulated control PBMCs (B), poly I:C stimulated PBMCs (C), non-poly I:C-stimulated control PBMCs (D), CpG ODN stimulated PBMCs (E), non-CpG stimulated control PBMCs (F). The color scale bar (white-orange-red) indicate increasing expression levels (low-medium-high).

**Fig 4:**
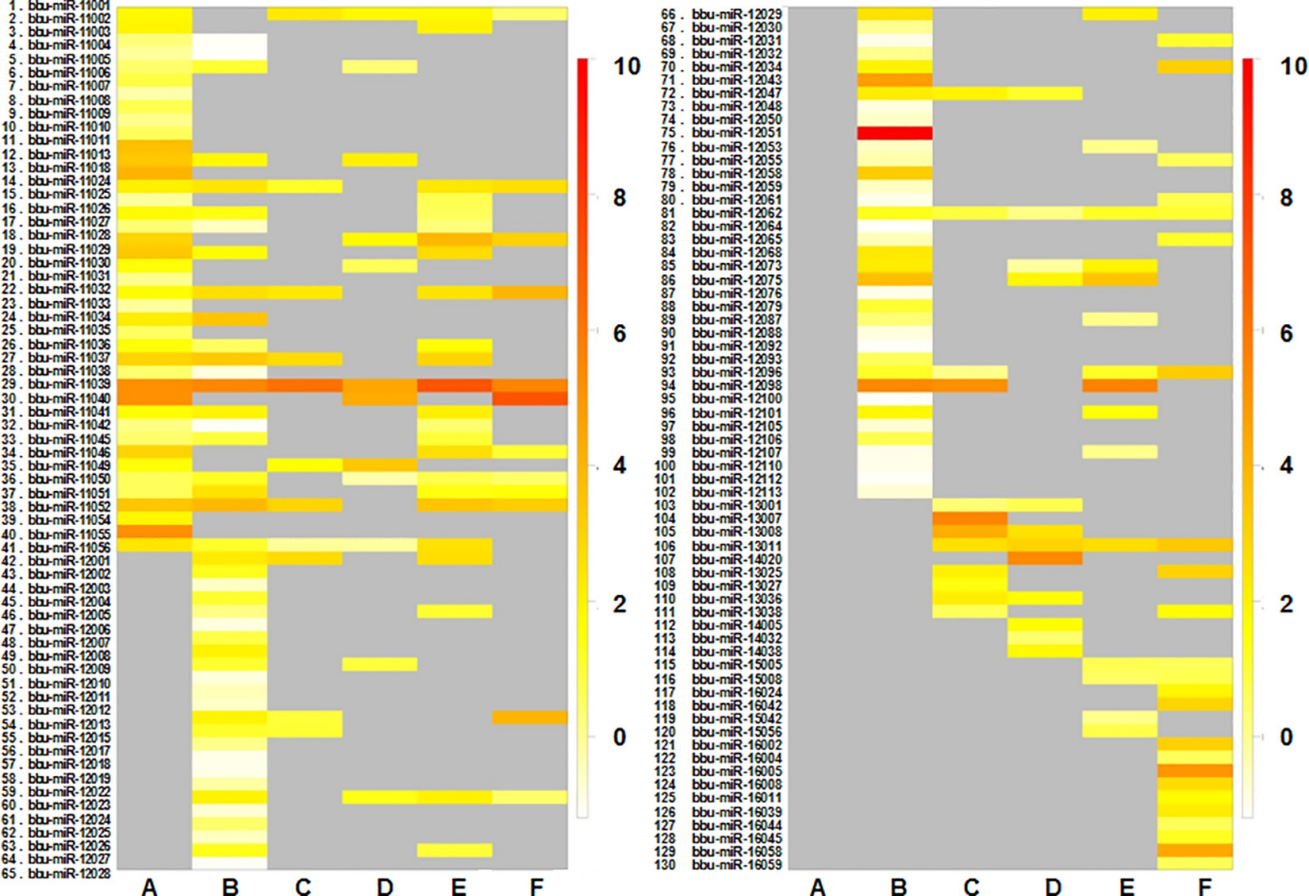
Heatmap of novel miRNA expression across the TLR stimulated and control samples. LPS stimulated PBMCs (A), non-LPS-stimulated control PBMCs (B), poly I:C stimulated PBMCs (C), non-poly I:C-stimulated control PBMCs (D), CpG ODN stimulated PBMCs (E), non-CpG stimulated control PBMCs (F). The color scale bar (white-orange-red) indicate increasing expression levels (low-medium-high).

The sample wise overall abundance of known and novel miRNA as log transformed values has been shown as box-plot (Fig 2). While the log expression levels of the each known (total 160) and the novel (total 130) miRNAs across the six samples are depicted as heatmap (Figs 3 and 4, respectively). The two miRNAs (bta-miR-103 and -191) were found to highly abundant in all the samples, while bta-miR-21-5p was highly and uniquely expressed in LPS treated PBMCs only. Other miRNAs which abundantly expressed in LPS-, CpG-treated and control samples include bta-miR-19b, -29b, -15a, -19a, -30d, -30b-5p and members of let family (let 7a-5p, let 7g & let 7f). Whereas in Poly I:C treated PBMCs, only three miRNAs (viz. bta-miR-191, -103 & -19b) showed more abundance (>7). Similarly, only three identified novel miRNAs across all the samples had abundance more than 7 viz. bta-miR-11039 (abundant in PolyI:C and CpG-treated PBMCs), bta-miR-11040 (abundant in CpG-treated PBMCs) and bta-miR-12051 (abundant in LPS non-treated PBMCs). Additionally, there is only one novel miRNA (bta-miR-11039) that is expressed in all the six samples (S5 Table).

**Fig 5:**
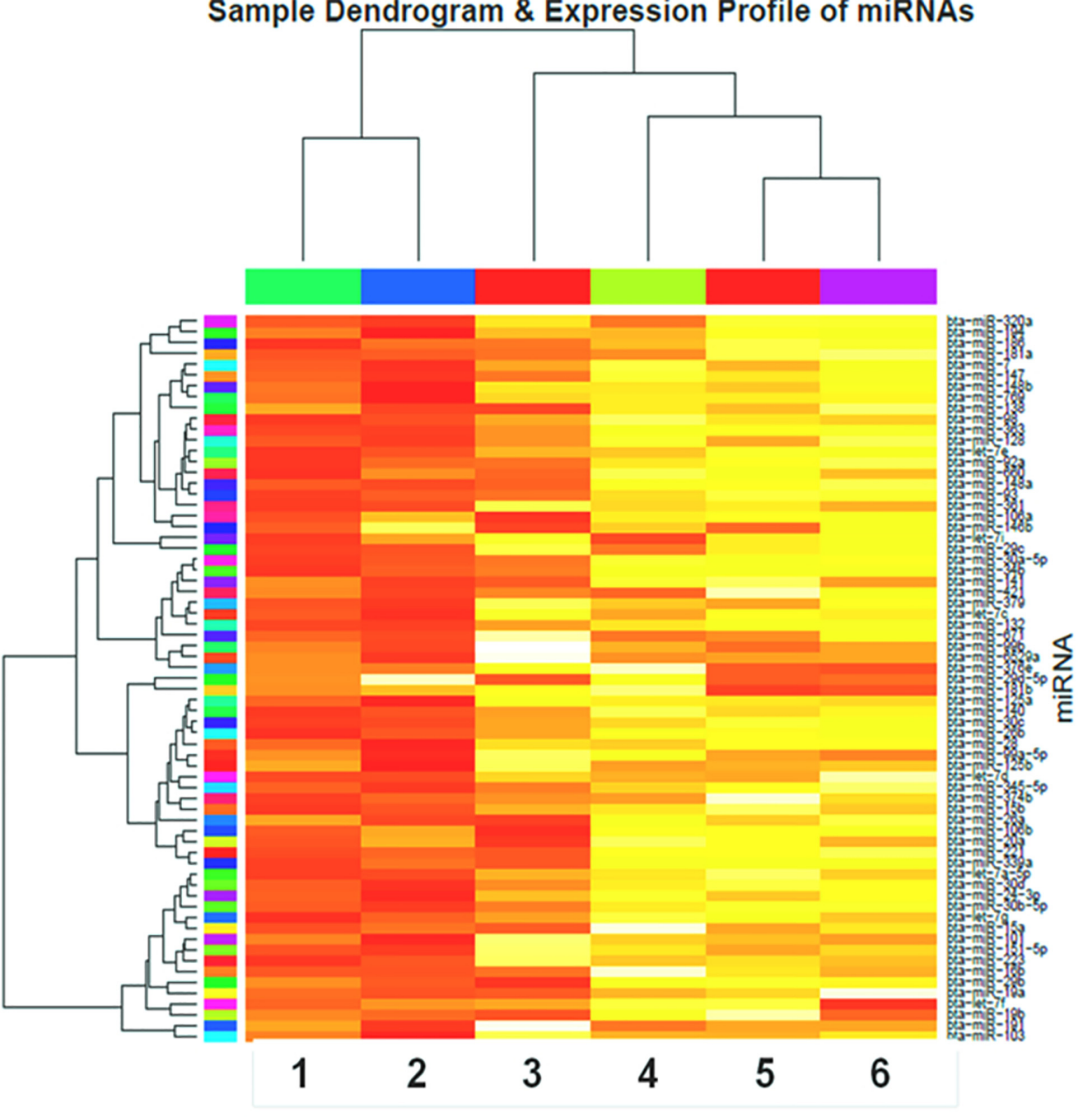
The sample dendrogram and expression profiles of known and novel miRNAs across TLR stimulated and control samples. CpG ODN stimulated PBMCs (A), non-LPS-stimulated control PBMCs (B), non-CpG stimulated control PBMCs (C), poly I:C stimulated PBMCs (D), LPS stimulated PBMCs (E), non-poly I:C-stimulated control PBMCs (F).

The sample dendrogram of the known and novel miRNAs expressed in all the six samples (Fig 5) indicated that two treatment groups (viz. LPS- and Poly I:C- treated samples) along with Poly I:C control group exhibited the most similar miRNA expression profiles. Moreover the miRNAs were also clustered together based on their expression levels, for example, miRNAs bta-miR-320a, -194, -186 and -181a; having similar expression profiles formed a cluster.

The differentially expressed known and novel miRNAs in treatment groups against their respective controls have been identified and the miRNAs which are up- or down-regulated in treatment groups with ratio more than 2 from their controls have been enlisted (S6 Table).

### Cross-validation of miRNA expression by qPCR

A total of six miRNAs that were identified to be differentially expressed among the different treatment versus control groups (i.e. 2 differentially expressed miRNAs per each group) was selected for verification by real time PCR using TaqMan microRNA assays. Relative quantification was performed by normalization of the expression of target miRNAs against that of miR-191, used as endogenous control. miR-191 has previously been reported to have stable expression in bovine serum [30] and in bovine oocytes and early embryos, and porcine oocytes [31] and thus is suitable as optimal endogenous expression normalizer for reverse transcription quantitative real-time polymerase chain reaction-based detection of microRNAs.

**Fig 6:**
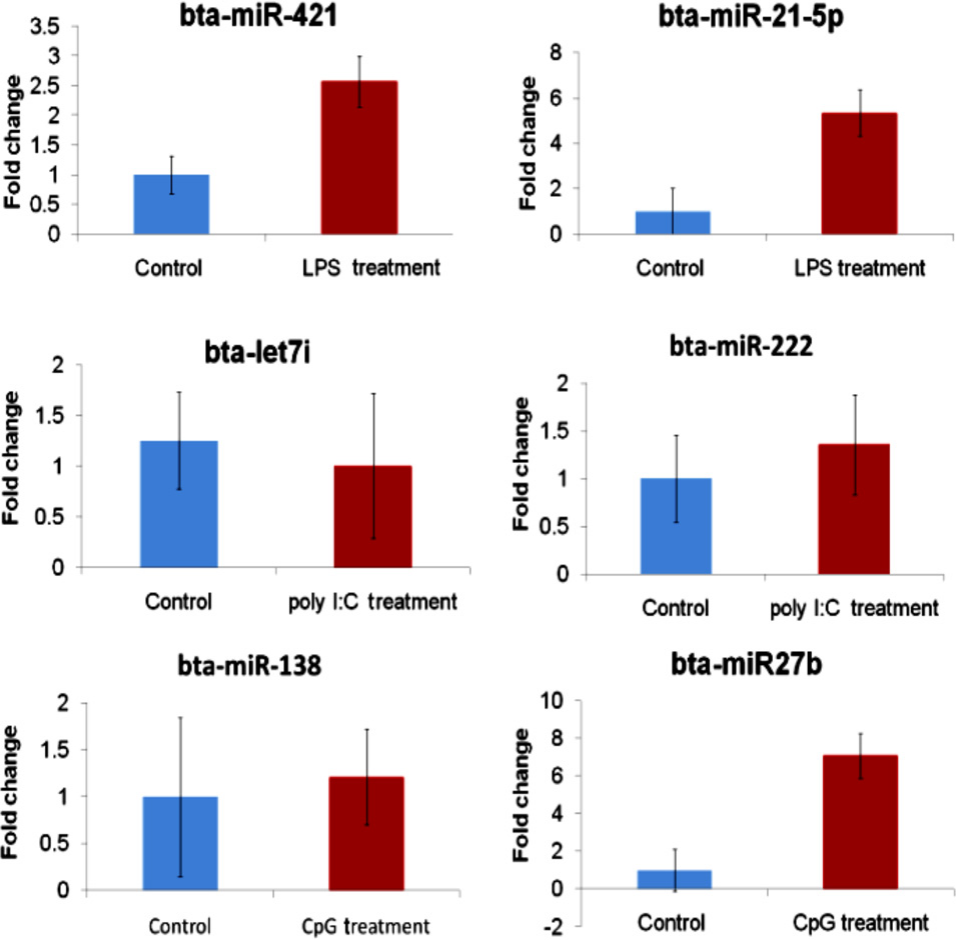
qRT PCR of six differentially expressed miRNAs across TLR stimulated and control samples by TaqMan miRNA assays.

The data presented in Fig 6 demonstrates that for most of miRNA studied by qRT-PCR, the expression pattern i.e. fold changes observed were in line with expression changes that were observed by RNA sequence data. In LPS stimulated PBMCs, qPCR results revealed 2.5 folds up expression of bta-mir-421 against the control as compared to a 19.5 fold upregulation revealed by sequencing. While bta-miR21-5p is found to be upregulated by 5.34 fold in LPS stimulated than control PBMCs. Further as per qPCR results bta-let7i was found to be down regulated by 1.25 folds in polyI:C stimulated PBMCs, as compared to the down regulation of 10.8 folds as revealed by sequencing experiment. Up regulation of bta-miR22 by 1.35 folds in polyI:C stimulated PBMCs was also detected. Finally, in CpG ODN stimulated PBMCs, TaqMan qPCR revealed 1.21 folds up regulation of bta-miR-138 in comparison to 2.44 folds uporegulation revealed by sequencing. bta-miR-27b was found to upregulated by 7.07folds in CpG stimulated PBMCs against control by qPCR.

### Gene ontology and functional classification

**Fig 7:**
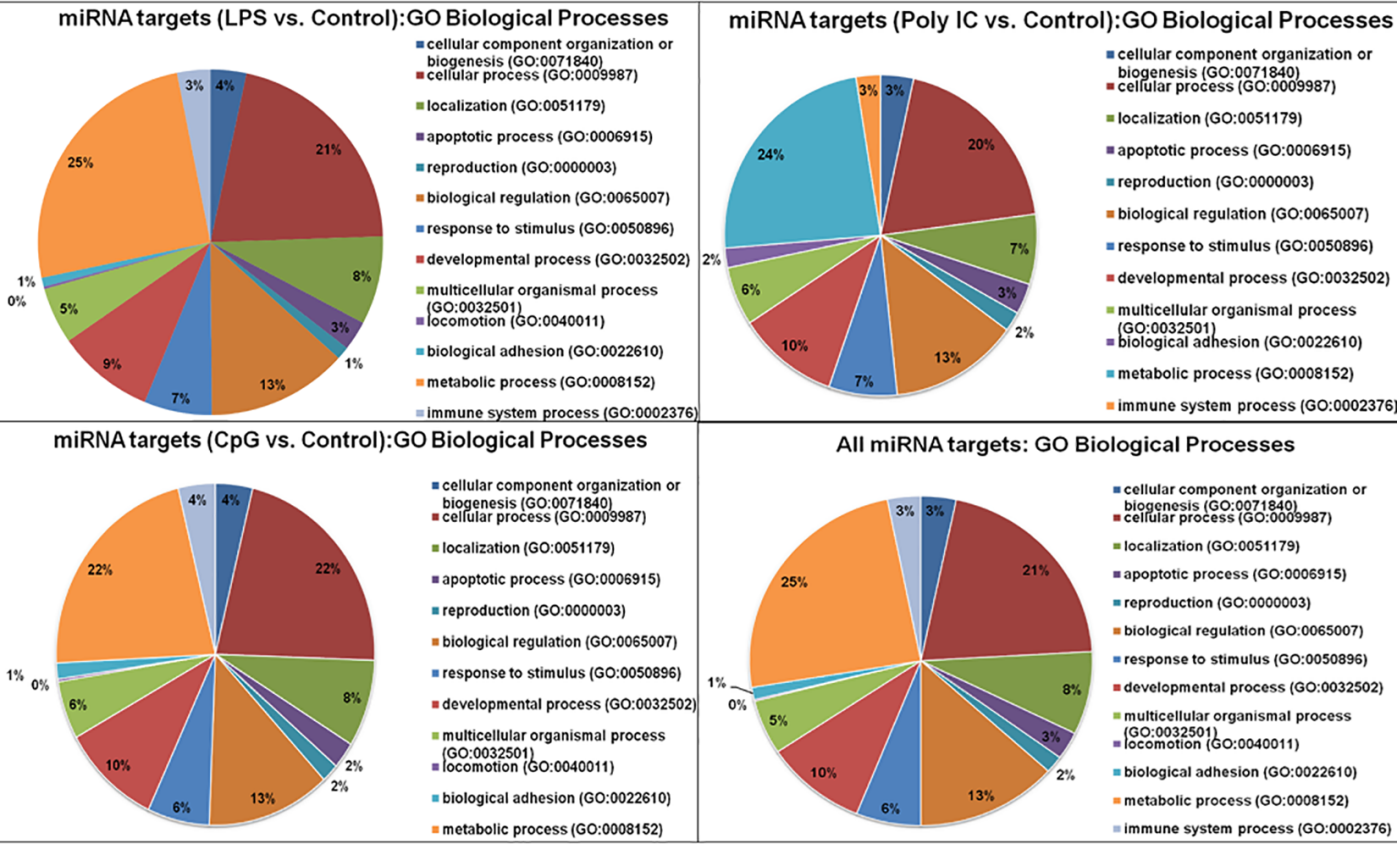
Piecharts representing the Gene Ontology (GO) based classification (www.pantherdb.org) of miRNA targets in various types of biological processes.

The top potential targets of miRNAs across the different treatment and control groups that are supported by all three tools (TargetScan, miRDB and PicTar) were selected (S7 Table). Gene ontology based classification of targets was performed for differentially expressed and uniquely expressed miRNA in treatment group against the control and predicted the involvement of targets in various biological processes. In case of LPS stimulated vs. control group, for 421 miRNA target genes a total of 834 biological process hits were predicted which was categorized into 13 groups (including cellular process, apoptotic process, biological regulation, response to stimulus, immune system process etc) (Fig 7A). Likewise for Poly I:C stimulated and control group, 354 process hits (falling in 12 categories) were predicted for 189 miRNA target genes (Fig 7B). While 399 process hits spanning 13 categories of biological processes were predicted for 216 targets of miRNAs belonging to CpG stimulated and control groups (Fig 7C). Finally all the 612 miRNA targets (of differentially expressed and uniquely expressed miRNAs across all the groups) were predicted to be involved in 1178 processes of 13 categories (Fig 7D).

## Discussion

Plethora of reports are available on immune-related miRNAs that have been identified to play role in host response to lipopolysaccharide (LPS) stimulation [32–33], which is frequently used to mimic a Gram-negative bacterial infection. Lawless and colleagues identified over 145 miRNAs that have been shown to be differentially expressed in response to LPS across multiple different species and tissues [34]. In the present study, a total of 18 miRNAs were found to be up-regulated on LPS stimulation of bubaline PBMCs. Out of these miRNAs,miR-15b (up-regulated 2 folds) have been previously reported to show up-regulation using real-time RT-PCR in dose- and time-dependent manner along with other miRNAs (let-7d, miR-16, miR-25, miR-92a, miR-103, miR-107 and miR-451) in the whole blood of C57BL/6 mice injected with LPS [35]. Similarly, miR-132, which is expressed 2.4 times greater in LPS challenged PBMCs than the control, have been initially identified and showed increased expression, along with miR-146and miR-155, in the human monocytic THP-1 cell line in response to lipo-polysaccharide (LPS) [14]. These researchers further provided evidence that miR-146a expression was not increased after in response to TLR-3, -7 or -9 ligands for the viral and bacterial nucleic acids. Another member of let-7 family, bta-let-7c, which was found to be up-regulated by 3.2 folds in the LPS challenged bubaline PBMCs of the present study have been previously reported [35] to be up-regulated in whole blood of mice 6 h following 100-μg LPS (L3755) injection. The bta-miR-186 was observed to be upregulated by 2.8 folds in the LPS treated PBMCs, while its expression was down regulated (19.38 folds) in CpG treated PBMCs; in the present study. In contrast, Hsieh and colleagues observed significant down-regulation of miR-186 in whole blood of C57BL/6 mice injected 100μg of LPS using an miRNA array [35].

In TLR3 ligand (CpG) challenged PBMCs of the present study, bta-miR-99b was found to be down-regulated (2-fold) with respect to its control. The miR-99b, known to inhibit the production of pro-inflammatory cytokines; had previously been reported to be expressed in unchallenged Bovine alveolar macrophages (BAMs) [36] and up-regulated in murine dendritic cells infected with *M*. *tuberculosis* [37]. Further, the miRNA bta-let7i that was observed to be maximally down-regulated miRNA in Poly I:C and CpG stimulated PBMCs (as compared to the respective control samples), has several well documented roles in immunity [7, 38–39]. Moreover, the miRNA-let7i along with other let-7 family members was observed to be down-regulated in murine macrophages during Salmonella infection [40]. The miRNA bta-let7i has also been found to be highly expressed in non-stimulated/ unchallenged Bovine alveolar macrophages (BAMs) [36].

The miR-29, originally identified as a repressor ofHIV-1 mRNA [41], in the present study (bta-miR29c), was observed to be up-regulated (4.4- fold) in LPS stimulated PBMCs while it was down-regulated in both PolyI:C stimulated (2- fold) and CpG stimulated PBMCs (13-fold). Previously miR-29 has been reported to be down-regulated in NK cells during systemic infection of mice with *Listeria monocytogenes* or *Mycobacterium bovis*, thus resulting in the enhanced expression of its target INF-γ [42]. Infection with *Mycobacterium avium* also induces mir-29a along with let-7e, in human macrophages which further targets the genes for caspase 7 and 3, respectively. In this way, miR 29a and let-7e controls the inhibition of apoptosis upon Mycobacteria infection [43].

More recently, Jin and colleagues profiled the global expression of miRNAs using RNA-Seq in bovine mammary epithelial cells challenged with and without heat-inactivated *S*. *aureus* or *E*. *coli* at different time intervals (0, 6, 12, 24, and 48 hrs) [44]. *E*. *coli* initiated an earlier regulation of miRNAs (with 6 miRNAs differentially regulated within the first 6 hrs post challenge as compared to 1 miRNA for *S*. *aureus*) while *S*. *aureus* presented a delayed response. Target gene and KEGG pathways predictions of pathogen differentially expressed miRNAs indicated a significant enrichment in gene ontology functional categories in development/cellular processes, cell growth and death, immune system, signal transduction, nervous system, and human diseases [44]. Furuse and coworkers identified first candidate microRNA of bacterial origin, a 23-nt sRNA bounded by RISC and derived from an RNA stem-loop, in mycobacterial species, *M*. *marinum*. Artificial over expression of this potential bacterial pre-microRNA in *in-vitro* infected cultured cells resulted in the efficient repression of a target mRNA [45]. Further, 9 miRNAs (mir-127-3p, mir-133a-1-3p, mir-133a-2-3p, mir-133a-1-5p, mir-133b-3p, mir-193b-3p, mir-215-5p, mir-434-3p, mir-676-3p) showed up-regulation greater than 4-fold (p-value < 0.01) in the serum of mice after gram-positive bacterial infection [46].

## Conclusion

Here we have provided the first atlas of miRNA expression in TLR ligand stimulated PBMCs of buffaloes, which will serve as a reference point for future functional studies or challenge experiments directed to uncover the role of miRNAs in immunity and disease pathogenesis.

## Supporting Information

S1 Table Quantity of sRNA and miRNA in each sample checked on Agilent’s 2100 Bioanalyzer. (DOCX)

S2 Table List of the known miRNAs (homologous to reported taurine miRNAs) identified in the bubaline PBMCs treated with TLR ligands vis-à-vis the respective control groups (DOCX)

S3 Table List of the novel miRNAs (with respect to taurine miRNAs) identified in the bubaline PBMCs treated with TLR ligands vis-à-vis the respective control groups (DOCX)

S4 Table List of the novel miRNAs that have hairpin sequences matched to the miRBase hairpin sequences (DOCX)

S5 Table List of the novel miRNAs (with respect to taurine miRNAs) expressed commonly in all the groups of treatment and control. (DOCX)

S6 Table List of known and novel miRNAs of TLR ligand stimulated and non-stimulated control samples having fold change more than 2. (DOCX)

S7 Table List of potential target genes of differentially and uniquely expressed miRNAs in TLR ligand stimulated and non-stimulated control samples. (DOC)
