# Supplementary material for: Identification of the MicroRNA Repertoire in TLR-Ligand Challenged Bubaline PBMCs as a Model of Bacterial and Viral Infection
Source: PLoS One. 2016 Jun 3;11(6):e0156598. doi: 10.1371/journal.pone.0156598 (PMC4892552; doi:10.1371/journal.pone.0156598)
Supplement: S1 Table — (DOCX) [file pone.0156598.s001.docx]

**S1 Table. Quantity of sRNA and miRNA in each sample checked on Agilent’s 2100 Bioanalyzer.**

|  | **Sample-1** | **Sample 2** | **Sample 3** | **Sample 4** | **Sample 5** | **Sample 6** |
| --- | --- | --- | --- | --- | --- | --- |
| **sRNA Conc. (pg/µl)** | 38,786.6 | 105,216.1 | 94,741.2 | 18,334.1 | 85,458.8 | 69,514.4 |
| **miRNA Conc. (pg/µl)** | 2,678.2 | 8,592.5 | 8,666.3 | 975.2 | 7,277.4 | 6,684.6 |
| **miRNA / sRNA Ratio (%)** | 7 | 8 | 9 | 5 | 9 | 10 |
