# Supplementary material for: Identification of the MicroRNA Repertoire in TLR-Ligand Challenged Bubaline PBMCs as a Model of Bacterial and Viral Infection
Source: PLoS One. 2016 Jun 3;11(6):e0156598. doi: 10.1371/journal.pone.0156598 (PMC4892552; doi:10.1371/journal.pone.0156598)
Supplement: S2 Table — (DOCX) [file pone.0156598.s002.docx]

**S 2Table. List of the known miRNAs (homologous to reported taurine miRNAs) identified in the bubaline PBMCs treated with TLR ligands vis-à-vis the respective control groups**

| **SN** | **miRNA*** | **Mature1_seq (5’-3)** | **Sequence (5’-3)** | **Position** | **miRNA**  **length** | **Hairpin**  **Length** | **HairpinGC** | **PVal**** |
| --- | --- | --- | --- | --- | --- | --- | --- | --- |
| 1 | bta-miR-125b | ucccugagacccuaacuuguga | cgcgcgccucucaaUCCCUGAGACCCUAACUUGUGAuguuuaccguuuaaauccacggguuaggcucuugggagcugcgagucgcgcc | 15 to 36 | 22 | 69 | 49.28 | 0.0099 |
| 2 | bta-miR-99a-5p | aacccguagauccgaucuugu | cccauuggcauaAACCCGUAGAUCCGAUCUUGUggugaaguggaccgcacaagcucgcuucuaugggucugugucagugug | 13 to 33 | 22 | 55 | 54.55 | 0.0099 |
| 3 | bta-let-7c | ugagguaguagguuguaugguu | gcauccggguUGAGGUAGUAGGUUGUAUGGUUuagaguuacacccugggaguuaacuguacaaccuucuagcuuuccuuggagc | 11 to 32 | 22 | 79 | 45.57 | 0.0099 |
| 4 | bta-miR-6529a | gagagaucagaggcgcagagu | cuguucucuuGAGAGAUCAGAGGCGCAGAGUgcgucaaugucaaugaagccugugccuuuuaccucuuuaagagcgcac | 11 to 31 | 21 | 75 | 49.33 | 0.0099 |
| 5 | bta-miR-28 | aaggagcucacagucuauugag | gguccuugcccucAAGGAGCUCACAGUCUAUUGAGuugccuuucugacuuucccacuagauugagagcuccuggagggcaggcacu | 14 to 35 | 22 | 76 | 51.32 | 0.0099 |
| 6 | bta-miR-15b | uagcagcacaucaugguuuaca | uugagaccuuaaaguacugUAGCAGCACAUCAUGGUUUACAuacuacagucaagaugcgaaucauuauuugcugcucuagaaauuuaaggaaauucau | 20 to 41 | 22 | 56 | 39.29 | 0.0099 |
| 7 | bta-miR-16b | uagcagcacguaaauauuggc | cauacuuguuccgcugUAGCAGCACGUAAAUAUUGGCguaguaaaauaaauauuaaacaccaauauuauugugcugcuuuagcgugacaggga | 17 to 37 | 23 | 78 | 35.90 | 0.0099 |
| 8 | bta-miR-147 | gugugcggaaaugcuucugcua | uaugaaucuaguggaaacacuucugcacaggcuagauuauggauaccaGUGUGCGGAAAUGCUUCUGCUAcauuuuuagg | 49 to 70 | 22 | 60 | 46.67 | 0.0297 |
| 9 | bta-miR-181a | aacauucaacgcugucggugaguu | ugagcuccgagguugcuucagugAACAUUCAACGCUGUCGGUGAGUUuggaauuaaaaaucaaaaccaucgaccguugauuguacccuauggccaaccaccaucuccacc | 24 to 47 | 23 | 72 | 48.61 | 0.0099 |
| 10 | bta-miR-181b | aacauucauugcugucgguggguu | cuugggcagagguucuuucuuaaaaggucacaaucAACAUUCAUUGCUGUCGGUGGGUUgaacuguguggacaagcucacugaacaaugagugcaacuguggccccgcau | 36 to 59 | 23 | 56 | 39.29 | 0.0099 |
| 11 | bta-miR-15a | uagcagcacauaaugguuugu | ccuuggaguaaagUAGCAGCACAUAAUGGUUUGUggauuuugaaaaggugcaggccauauugugcugccucaaaaauacaagg | 14 to 34 | 21 | 59 | 45.76 | 0.0099 |
| 12 | bta-miR-19a | ugugcaaaucuaugcaaaacuga | gcaguccucuguuaguuuugcauaguugcacuacaagaagaauguaguUGUGCAAAUCUAUGCAAAACUGAugguggccugc | 49 to 71 | 22 | 72 | 38.89 | 0.0099 |
| 13 | bta-miR-20a | uaaagugcuuauagugcagguag | guagcacUAAAGUGCUUAUAGUGCAGGUAGuguuuaguuaucuacugcauuaugagcacuuaaaguacugc | 8 to 30 | 23 | 71 | 38.03 | 0.0099 |
| 14 | bta-miR-19b | ugugcaaauccaugcaaaacuga | cacuguucuaugguuaguuuugcagguuugcauccagcugugugauauucugcUGUGCAAAUCCAUGCAAAACUGAcugugguagug | 54 to 76 | 22 | 75 | 44.00 | 0.0099 |
| 15 | bta-miR-92a | uauugcacuugucccggccugu | cuuucuacacagguugggaucgguugcaaugcuguguuucuguauggUAUUGCACUUGUCCCGGCCUGUugaguuugg | 48 to 69 | 22 | 61 | 50.82 | 0.0099 |
| 16 | bta-miR-151-5p | ucgaggagcucacagucuagu | ccugcccUCGAGGAGCUCACAGUCUAGUacgucucauccccuacuagacugaagcuccuugaggacagg | 8 to 28 | 21 | 69 | 56.52 | 0.0099 |
| 17 | bta-miR-30d | uguaaacauccccgacuggaagcu | guuguUGUAAACAUCCCCGACUGGAAGCUguaccacacagcuaagcuuucagucagauguuugcugcuac | 6 to 29 | 22 | 58 | 48.28 | 0.0099 |
| 18 | bta-miR-30b-5p | uguaaacauccuacacucagcu | ccaaguuuucaguucaUGUAAACAUCCUACACUCAGCUguaacacacgagucggcugggagguggauguuuacuucagcugacuugga | 17 to 38 | 22 | 56 | 50.00 | 0.0099 |
| 19 | bta-miR-34b | aggcaguguaauuagcugauug | gugcucgguuuguAGGCAGUGUAAUUAGCUGAUUGuacucucaugcuuacaaucacuaguuccacugccaucaaaacaaggcac | 14 to 35 | 23 | 55 | 43.64 | 0.0099 |
| 20 | bta-let-7a-5p | ugagguaguagguuguauaguu | ugggaUGAGGUAGUAGGUUGUAUAGUUuuagggucacacccaccacugggagauaacuauacaaucuacugucuuuccua | 6 to 27 | 22 | 79 | 44.30 | 0.0099 |
| 21 | bta-miR-194 | uguaacagcaacuccaugugga | uucuuaacggcgucaucgauUGUAACAGCAACUCCAUGUGGAcugugcgucaauuuccaguggagaugcuguuacuuuugauggcugccaauucacu | 21 to 42 | 21 | 70 | 44.29 | 0.0099 |
| 22 | bta-miR-29b | uagcaccauuugaaaucaguguu | cuucaggaagcugguuucauauggugguuuagauuuaaauagugauugucUAGCACCAUUUGAAAUCAGUGUUcuuggggg | 51 to 73 | 23 | 81 | 39.51 | 0.0099 |
| 23 | bta-miR-29d-5p | ugaccgauuucuccugguguu | aucucuuacacaggcUGACCGAUUUCUCCUGGUGUUcagagucuguuuuugucuagcaccauuugaaaucgauuaugauguaggggga | 16 to 36 | 21 | 57 | 56.14 | 0.0396 |
| 24 | bta-miR-29c | uagcaccauuugaaaucgguua | aucucuuacacaggcugaccgauuucuccugguguucagagucuguuuuugucUAGCACCAUUUGAAAUCGGUUAugauguaggggga | 54 to 75 | 22 | 58 | 43.10 | 0.0099 |
| 25 | bta-miR-138 | agcugguguugugaaucaggccg | cuggcacggugcgguggggcAGCUGGUGUUGUGAAUCAGGCCGucgccaaucagagaacggcuacuucacaacaccagggucacaccccaccccagg | 21 to 43 | 23 | 81 | 56.79 | 0.0099 |
| 26 | bta-miR-140 | uaccacaggguagaaccacgga | ucucucuguguccugccagugguuuuacccuaugguagguuacgucaugcuguucUACCACAGGGUAGAACCACGGAcaggauaccggggcacc | 56 to 78 | 22 | 78 | 51.28 | 0.0099 |
| 27 | bta-miR-769 | ugagaccuccggguucugagcu | gacuuggugcugauucccggcgcucugaccUGAGACCUCCGGGUUCUGAGCUgugauguugcuucccagcugggaucucuggggucucgguucagggucgggaccucuggguucugagc | 31 to 52 | 22 | 71 | 59.15 | 0.0099 |
| 28 | bta-miR-99b | cacccguagaaccgaccuugcg | ggcaccCACCCGUAGAACCGACCUUGCGgggccuucgccgcacacaagcucgugucuguggguccguguc | 7 to 28 | 22 | 70 | 67.14 | 0.0297 |
| 29 | bta-let-7e | ugagguaggagguuguauagu | cccgggcUGAGGUAGGAGGUUGUAUAGUugaggaggacacccaaggagaucacuauacggccuccuagcuuuccccagg | 8 to 28 | 21 | 73 | 54.79 | 0.0099 |
| 30 | bta-miR-125a | ucccugagacccuuuaaccugug | ugccggccucugcgUCCCUGAGACCCUUUAACCUGUGaggacguccagggucacaggugagguucuugggagccuggcguccggcc | 15 to 37 | 22 | 57 | 56.14 | 0.0396 |
| 31 | bta-miR-132 | uaacagucuacagccauggucg | ccgcccccgcgucuccagggcaaccguggcuuucgauuguuacugugggaaccggaggUAACAGUCUACAGCCAUGGUCGccccgcagcacgcccacgcgc | 59 to 79 | 22 | 66 | 57.58 | 0.0099 |
| 32 | bta-miR-128 | ucacagugaaccggucucuuu | ugagcuguuggauucggggccguagcacugucugagagguuuacauuucUCACAGUGAACCGGUCUCUUUuucagcugcuuc | 50 to 70 | 21 | 70 | 50.00 | 0.0099 |
| 33 | bta-miR-26b | uucaaguaauucaggauagguu | ugcccgggacccagUUCAAGUAAUUCAGGAUAGGUUgugugcuguccagccuguucuccauuacuuggcucgggggccggugccc | 15 to 36 | 22 | 58 | 44.83 | 0.0099 |
| 34 | bta-miR-103 | agcagcauuguacagggcuauga | cugcccucggcuucuuuacagugcugccuuguugcauauggaucaAGCAGCAUUGUACAGGGCUAUGAaggc | 46 to 68 | 23 | 75 | 53.33 | 0.0198 |
| 35 | bta-miR-7 | uggaagacuagugauuuuguuguu | guggagcagccagccccaucUGGAAGACUAGUGAUUUUGUUGUUgucuccugcgcucaacaacaagucccagucugccgcauggugcuggccaccgca | 21 to 44 | 23 | 57 | 49.12 | 0.0198 |
| 36 | bta-miR-345-5p | gcugacuccuaguccagugcu | acccaaacccaggucuGCUGACUCCUAGUCCAGUGCUugugauggcuggugggcccugaacuaggggucuggaggccuggguuugaauauc | 17 to 37 | 21 | 60 | 58.33 | 0.0099 |
| 37 | bta-miR-379 | ugguagacuauggaacguagg | ugguuccugcagagaUGGUAGACUAUGGAACGUAGGcuuugugauuuuugaccuauguaacaugguccacuaacucucaguaucca | 16 to 36 | 21 | 70 | 42.86 | 0.0099 |
| 38 | bta-miR-376e | aacauagaggaaaauccacauu | ugauauucaaaagguggauauuccuucuauguuuacaggauugacagcuaAACAUAGAGGAAAAUCCACAUUuuaaguaucu | 51 to 72 | 22 | 78 | 30.77 | 0.0099 |
| 39 | bta-miR-26a | uucaaguaauccaggauaggcu | aaggccguggccucgUUCAAGUAAUCCAGGAUAGGCUgugcaggucccaaggggccuauucucgguuacuugcacgcggacgcgggccug | 16 to 37 | 22 | 69 | 56.52 | 0.0099 |
| 40 | bta-let-7g | ugagguaguaguuuguacaguu | aggcUGAGGUAGUAGUUUGUACAGUUugagggucuaugauaccacccgguacaggagauaacuguacaggccacugccuugcc | 5 to 26 | 22 | 90 | 52.22 | 0.0099 |
| 41 | bta-miR-191 | caacggaaucccaaaagcagcug | ggcuggacagcgggCAACGGAAUCCCAAAAGCAGCUGuugucuccagagcauuccagcugcgcuuggauuucguucccugcucuccugccu | 15 to 37 | 22 | 74 | 55.41 | 0.0099 |
| 42 | bta-miR-106b | uaaagugcugacagugcagau | ccugccggggcUAAAGUGCUGACAGUGCAGAUagugguccugugugcuaccgcacuguggguacuugcugcuccggcagg | 12 to 32 | 21 | 72 | 59.72 | 0.0099 |
| 43 | bta-miR-93 | caaagugcuguucgugcaggua | cugggggcucCAAAGUGCUGUUCGUGCAGGUAgugugaucaccugaccuacugcugagccagcacuucccgagcccc | 11 to 32 | 23 | 74 | 60.81 | 0.0099 |
| 44 | bta-miR-339a | ucccuguccuccaggagcucac | ggggcagccgcugUCCCUGUCCUCCAGGAGCUCACuugguccggccgugcgcuccucgaggccagagcccgugucugc | 14 to 35 | 21 | 65 | 70.77 | 0.0396 |
| 45 | bta-miR-146b | ugagaacugaauuccauaggcugu | uaagagaacuuuggccaccuggcucUGAGAACUGAAUUCCAUAGGCUGUgagcucuagcaaaugcccuagggacucaguucuggugcccggcugugcuacaccauc | 26 to 49 | 22 | 68 | 52.94 | 0.0099 |
| 46 | bta-miR-186 | caaagaauucuccuuuugggcu | ugcuuauaacuuucCAAAGAAUUCUCCUUUUGGGCUuucugauuuuauuuuaagcccaaaggugaauuuuuugggaaguuugagcu | 15 to 36 | 22 | 73 | 32.88 | 0.0099 |
| 47 | bta-miR-30c | uguaaacauccuacacucucagc | cagacuguaaccaugccguagugugUGUAAACAUCCUACACUCUCAGCugugagcucgagguggcugggagaggguuguuuacuccuucugccauggaaaacguc | 26 to 48 | 22 | 57 | 52.63 | 0.0099 |
| 48 | bta-miR-148a | ucagugcacuacagaacuuugu | gaggcaaaguucugagacacuccgacucugaauaugauagaagUCAGUGCACUACAGAACUUUGUcuc | 44 to 65 | 22 | 68 | 44.12 | 0.0099 |
| 49 | bta-miR-671 | aggaagcccuggaggggcuggag | gccgccgaccuggcaggccaggaagaggAGGAAGCCCUGGAGGGGCUGGAGgugauggauguuuuccuccgguucucagggcuccaccuuuuccgggccguggagccagggcuggugc | 29 to 51 | 23 | 72 | 58.33 | 0.0099 |
| 50 | bta-miR-148b | ucagugcaucacagaacuuugu | uuagcauuugaggugaaguucuguuauacacucaggcuguggcucucugaaagUCAGUGCAUCACAGAACUUUGUcucgaaagcuuucua | 54 to 75 | 22 | 75 | 44.00 | 0.0198 |
| 51 | bta-let-7i | ugagguaguaguuugugcuguu | cuggcUGAGGUAGUAGUUUGUGCUGUUggucggguugugacauugcccgcuguggagauaacugcgcaagcuacugccuugcua | 6 to 27 | 22 | 91 | 56.04 | 0.0099 |
| 52 | bta-miR-141 | uaacacugucugguaaagaugg | gaccggcucuggguccaucuuccagcacaguguuggauggucuaauggugaagcuccUAACACUGUCUGGUAAAGAUGGcccccggcugg | 58 to 79 | 22 | 84 | 55.95 | 0.0099 |
| 53 | bta-miR-24-3p | uggcucaguucagcaggaacag | cucugccucccgugccuacugagcugaaacacaguugauuugugcacacUGGCUCAGUUCAGCAGGAACAGg | 50 to 71 | 22 | 68 | 52.94 | 0.0099 |
| 54 | bta-miR-101 | uacaguacugugauaacugaa | aggcugcccuggcucaguuaucacagugcugaugcuguccauucuaaaggUACAGUACUGUGAUAACUGAAggauggcagcca | 51 to 71 | 21 | 70 | 40.00 | 0.0099 |
| 55 | bta-miR-320a | aaaagcuggguugagagggcga | cccgcggcgucucgcuccccuccgccuucucuucccgguucuucccggagucgggAAAAGCUGGGUUGAGAGGGCGAaaaag | 56 to 77 | 22 | 65 | 60.00 | 0.0099 |
| 56 | bta-let-7f | ugagguaguagauuguauaguu | ucagagUGAGGUAGUAGAUUGUAUAGUUgugggguagugauuuuacccuguucaggagauaacuauacaaucuauugccuucccuga | 7 to 28 | 22 | 87 | 40.23 | 0.0099 |
| 57 | bta-let-7d | agagguaguagguugcauaguu | ccuaggaAGAGGUAGUAGGUUGCAUAGUUuucgggcagggauuuugcccacaaggagguaacuauacgaccugcugccuuucuuagg | 8 to 29 | 22 | 87 | 49.43 | 0.0099 |
| 58 | bta-miR-30a-5p | uguaaacauccucgacuggaagcu | cUGUAAACAUCCUCGACUGGAAGCUgugaggcugcagaaaggcuuucagucggauguuugcagcugc | 2 to 25 | 23 | 74 | 55.41 | 0.0099 |
| 59 | bta-miR-363 | auugcacgguauccaucugcg | uguugucggguggaucacgaugcaauuuugauuaguauaauaggagaaaaAUUGCACGGUAUCCAUCUGCGaac | 51 t0 71 | 22 | 81 | 43.21 | 0.0099 |
| 60 | bta-miR-106a | aaaagugcuuacagugcaggua | ccuuggccauguAAAAGUGCUUACAGUGCAGGUAgcuuuuugagaucuacugcaaugcaagcacuucuuacauuaccaugg | 13 to 34 | 23 | 63 | 39.68 | 0.0099 |
| 61 | bta-miR-361 | uuaucagaaucuccagggguac | ggagcUUAUCAGAAUCUCCAGGGGUACuuauaauuugaaaaagucccccaggugugauucugauuugcuuc | 6 to 27 | 22 | 73 | 43.84 | 0.0099 |
| 62 | bta-miR-374b | auauaauacaaccugcuaagug | gaagaaauccuacucggauggAUAUAAUACAACCUGCUAAGUGuccuagcacuuaucagguuguauuaucauuguccgugucuauggcucucguc | 22 to 43 | 22 | 64 | 40.63 | 0.0099 |
| 63 | bta-miR-421 | aucaacagacauuaauugggcgc | gcacauuguaggccucauuaaauguuuguugaaugaaaaaaugaaucAUCAACAGACAUUAAUUGGGCGCcugcucugugaucuc | 48 to 70 | 23 | 67 | 37.31 | 0.0099 |
| 64 | bta-miR-660 | uacccauugcauaucggagcug | cugcuccuucucccgUACCCAUUGCAUAUCGGAGCUGugaauucucaaagcaccuccuaugugcauggauuacaggaggg | 16 to 37 | 22 | 69 | 49.28 | 0.0099 |
| 65 | bta-miR-98 | ugagguaguaaguuguauuguu | aggacucugcucaugcuggggUGAGGUAGUAAGUUGUAUUGUUgugggguagggauuuuaggccccaauuugaagauaacuauacaacuuacuacuuucccugguguguagcacauuca | 22 to 43 | 22 | 91 | 42.86 | 0.0099 |
| 66 | bta-miR-223 | ugucaguuugucaaauacccca | cccagccuccugcagugccaugcuccguguauuugacaagcugaguuggacacuccauguaguagUGUCAGUUUGUCAAAUACCCCAaguguggcauaugccuagcag | 66 to 87 | 22 | 77 | 46.75 | 0.0099 |
| 67 | bta-miR-221 | agcuacauugucugcuggguuu | ccaacauccaggucuagggcaugaaccuggcauacaauguagauuucuguguuuguugagcaacAGCUACAUUGUCUGCUGGGUUUcaggcuaccuggaaacacguucuu | 65 to 86 | 22 | 64 | 42.19 | 0.0099 |
| 68 | bta-miR-2284x | ugaaaaguucguucggguuuu | ggauuuaguauuggguuggcUGAAAAGUUCGUUCGGGUUUUuccauaagaacucaaauaaacuuuuuggccaacccaguaauaacacugaugcacc | 21 to 41 | 21 | 72 | 38.89 | 0.0099 |
| 69 | bta-miR-155 | uuaaugcuaaucgugauaggggu | ugUUAAUGCUAAUCGUGAUAGGGGUuuuuaccucggacugacuccuacauguuagcauuaaca | 3 to 25 | 23 | 75 | 45.33 | 0.0099 |
| 70 | bta-miR-7859 | aaaaacuggcagcuucauguaa | augugaaauuaccaguauuugucugcuuugaccuacAAAAACUGGCAGCUUCAUGUAA | 37 to 58 | 22 | 74 | 40.54 | 0.0198 |
| 71 | bta-miR-126-3p | cguaccgugaguaauaaugcg | ugacgggacauuauuacuuuugguacgcgcugugacacuucaaacuCGUACCGUGAGUAAUAAUGCGcuguca | 47 to 67 | 22 | 73 | 45.21 | 0.0099 |
| 72 | bta-miR-16a | uagcagcacguaaauauuggug | gucagcagugccuUAGCAGCACGUAAAUAUUGGUGuuaagauucuaaaauuaucuccaguauuaacugugcugcugaaguaagguuggc | 14 to 35 | 22 | 73 | 35.62 | 0.0099 |
| 73 | bta-miR-1388-3p | aucucagguuugucagcccgca | ccugggcggugccuucaggacuguccaaccugagaauggugagcauccagggacaAUCUCAGGUUUGUCAGCCCGCAaggugccguccccuc | 56 to 77 | 22 | 77 | 57.14 | 0.0198 |
| 74 | bta-miR-133a | uuugguccccuucaaccagcug | ugggaccgaaugcuuugcuaaagcugguaaaauggaaccaaaucaacuguucgauggaUUUGGUCCCCUUCAACCAGCUGuagcugcgcauugau | 59 to 80 | 22 | 76 | 46.05 | 0.0099 |
| 75 | bta-miR-296-5p | gagggccccccccaauccu | aggacccuuccgGAGGGCCCCCCCCAAUCCUguugugcuugguucagaggguugggcggaggcuuuccugaagggucu | 13 to 31 | 21 | 69 | 63.77 | 0.0198 |
| 76 | bta-miR-34c | aggcaguguaguuagcugauug | agucuaguuacuAGGCAGUGUAGUUAGCUGAUUGcuaauaauaccaaucacuaaccacacggccagguaaaaagauu | 13 to 34 | 22 | 63 | 41.27 | 0.0099 |
| 77 | bta-miR-139 | ucuacagugcacgugucuccagu | guguacUCUACAGUGCACGUGUCUCCAGUguggcucggaggcuggagacgcggcccuguuggaguaac | 7 to 29 | 23 | 59 | 64.41 | 0.0099 |
| 78 | bta-miR-326 | ccucugggcccuuccuccag | cucgucugucuguugggcuggaggcagggccuuugugaaggcggguugugcucagaucgCCUCUGGGCCCUUCCUCCAGcccagaggcggauuca | 60 to 79 | 21 | 77 | 64.94 | 0.0099 |
| 79 | bta-miR-129-3p | aagcccuuaccccaaaaagcau | cugcccuucgcgaaucuuuuugcggucugggcuugcuguacauaacucaauagccggAAGCCCUUACCCCAAAAAGCAUucgcggagggcgcac | 58 to 79 | 22 | 82 | 51.22 | 0.0099 |
| 80 | bta-miR-215 | augaccuaugaauugacagaca | uguacaggaaaAUGACCUAUGAAUUGACAGACAacgugacuaagucugucugucauuucuguaggccaauguucuguau | 12 to 33 | 22 | 56 | 42.86 | 0.0099 |
| 81 | bta-miR-34a | uggcagugucuuagcugguugu | ggccagcugugaguguuucuuUGGCAGUGUCUUAGCUGGUUGUugugaguaauaaugcaggaagcaaucagcaaguauacugcccuagaagugcugcacguuguggg | 22 to 43 | 22 | 76 | 42.11 | 0.0099 |
| 82 | bta-miR-130b | cagugcaaugaugaaagggcau | ggccugccugacacucuuucccuguugcacuacugugcgccccuggcaagCAGUGCAAUGAUGAAAGGGCAUcggucaggcc | 51 to 72 | 22 | 76 | 57.89 | 0.0198 |
| 83 | bta-miR-185 | uggagagaaaggcaguuccuga | gggggugagggacUGGAGAGAAAGGCAGUUCCUGAugguccccuccccaggggcuggcuuuccuccggccccuccuucc | 14 to 35 | 22 | 68 | 66.18 | 0.0099 |
| 84 | bta-miR-1306 | ccaccuccccugcaaacgucc | CCACCUCCCCUGCAAACGUCCagugaugcagagguaauggacguuggcucugguggug | 1 to 21 | 21 | 67 | 56.72 | 0.0099 |
| 85 | bta-miR-330 | gcaaagcacacggccugcagaga | cuucggcgaucacugccucucugggccugugucuuaggcucugcaagaucaaccgaGCAAAGCACACGGCCUGCAGAGAggcagcgcucagcuc | 57 to 79 | 23 | 66 | 59.09 | 0.0099 |
| 86 | bta-miR-21-5p | uagcuuaucagacugauguugacu | ugucgggUAGCUUAUCAGACUGAUGUUGACUguugaaucucauggcaacagcagucgaugggcugucugaca | 8 to 31 | 22 | 72 | 48.61 | 0.0099 |
| 87 | bta-miR-2331-3p | acccugcagccaaagaagcua | ggguggcuuccgugccugcagaugucugugaauuccucaaggcugagACCCUGCAGCCAAAGAAGCUAccc | 48 to 68 | 21 | 71 | 57.75 | 0.0099 |
| 88 | bta-miR-365-3p | uaaugccccuaaaaauccuuau | agagugcucgaggacagcaagaaaaaugagggacuuucaggggcagcuguguuuucugacucagucaUAAUGCCCCUAAAAAUCCUUAUuguucuugcagugugcaucagg | 68 to 89 | 22 | 78 | 39.74 | 0.0099 |
| 89 | bta-miR-423-5p | ugaggggcagagagcgagacuuu | auaaaggaaguuaggcUGAGGGGCAGAGAGCGAGACUUUucuauuuuccaaaagcucggucugaggccccucagucuugcuuccuaccccgcgc | 17 to 40 | 22 | 63 | 55.56 | 0.0099 |
| 90 | bta-miR-22-5p | aguucuucaguggcaagcuuua | ggcugagccgcaguAGUUCUUCAGUGGCAAGCUUUAuguccugacccagcuaaagcugccaguugaagaacuguugcccucugcc | 15 to 36 | 22 | 60 | 46.67 | 0.0099 |
| 91 | bta-miR-195 | uagcagcacagaaauauuggca | agcuccccuggcucUAGCAGCACAGAAAUAUUGGCAcugggaagaaagccugccaauauuggcugugcugcuccaggcaggguggug | 15 to 36 | 22 | 57 | 49.12 | 0.0099 |
| 92 | bta-miR-324 | cgcauccccuagggcauuggugu | aacuggcuaugccucccCGCAUCCCCUAGGGCAUUGGUGUaaagcuggagacccacugccccaggugcugcuggggguuguagucugac | 18 to 40 | 23 | 61 | 65.57 | 0.0990 |
| 93 | bta-miR-497 | cagcagcacacugugguuugua | ccaccccaguccugcucccgcccCAGCAGCACACUGUGGUUUGUAcggcacuguggccacguccaaaccacacugugguguuagagcgagggugggggaggcaccgcugagg | 24 to 45 | 21 | 82 | 62.20 | 0.0198 |
| 94 | bta-miR-744 | ugcggggcuagggcuaacagca | gguugggcggggUGCGGGGCUAGGGCUAACAGCAggcucacugacgguuucccggaaaccacgcacaugcuguugccacuaaccucaaccuuacucgguc | 13 to 34 | 22 | 90 | 60.00 | 0.0396 |
| 95 | bta-miR-10a | uacccuguagauccgaauuugug | gaucugucugucuucuguauaUACCCUGUAGAUCCGAAUUUGUGuaaggaauuuugugaucacaaauucguaucuaggggaauauguaguugacauaaacacuccgcuc | 22 to 44 | 22 | 56 | 39.29 | 0.0099 |
| 96 | bta-miR-338 | uccagcaucagugauuuuguuga | gcacgggccguccuccccaacaauauccuggugcugagugaugacacacgcaacUCCAGCAUCAGUGAUUUUGUUGAagagggcagcugcca | 55 to 77 | 22 | 75 | 50.67 | 0.0099 |
| 97 | bta-miR-375 | uuuuguucguucggcucgcguga | ccccgcgacgagccccucgcacaaaccggaccugagcgUUUUGUUCGUUCGGCUCGCGUGAggc | 39 to 61 | 22 | 72 | 69.44 | 0.0198 |
| 98 | bta-miR-582 | uuacaguuguucaaccaguuacu | aucuguucucuuuggUUACAGUUGUUCAACCAGUUACUaaucuaccuaauuguaaccaguugaacaacugaacccaaagggugcaaagugaaaacauu | 16 to 38 | 22 | 61 | 37.70 | 0.0099 |
| 99 | bta-miR-449a | uggcaguguauuguuagcuggu | ugugugauggguUGGCAGUGUAUUGUUAGCUGGUugaauaugugagugccaucagcuaacaugcaacugcuaucuuauugcauguaua | 13 to 34 | 22 | 60 | 43.33 | 0.0099 |
| 100 | bta-miR-211 | uucccuuugucauccuuugcc | uccccuggcugugugaccugugggcUUCCCUUUGUCAUCCUUUGCCcaggguucugaguggggcagggacagcaaaggggugcucagucgucaccucccacagcauggag | 26 to 46 | 21 | 66 | 59.09 | 0.0198 |
| 101 | bta-miR-380-3p | uauguaaugugguccacgucu | aagaugguugaccauagaacaugcgcugucucuaugucgUAUGUAAUGUGGUCCACGUCUu | 40 to 60 | 21 | 61 | 44.26 | 0.0099 |
| 102 | bta-miR-494 | ugaaacauacacgggaaaccuc | ucgauacuugaaggagagguuauccguguugucuucucuuuauuuaugaUGAAACAUACACGGGAAACCUCuuuuuuaguaucaa | 50 to 71 | 23 | 72 | 36.11 | 0.0099 |
| 103 | bta-miR-495 | aaacaaacauggugcacuucuu | uggugccuggaaagaaguugcccauguucuuuucgcugauaugugacgAAACAAACAUGGUGCACUUCUUuuccggcauca | 49 to 70 | 22 | 75 | 45.33 | 0.0099 |
| 104 | bta-miR-376a | aucauagaggaaaauccacgu | aauccuucuuugguauuuaaaagguagauucuccuucuaugaguacauuauuuacgauuaAUCAUAGAGGAAAAUCCACGUuuucaguaucaaaugcug | 61 to 81 | 21 | 76 | 28.95 | 0.0099 |
| 105 | bta-miR-376d | aucauagaggaaaauccacau | gguauuuaaaagguagauuuuccuucuaugauuacggguuuggugauuaAUCAUAGAGGAAAAUCCACAUuuucgguaucaa | 50 to 70 | 21 | 56 | 35.71 | 0.0099 |
| 106 | bta-miR-376b | aucauagaggaaaauccauguu | uucuuugguauuuaaaagguggauauuccuucuauguuuacgugauucauagauaAUCAUAGAGGAAAAUCCAUGUUuucaguaucaaaugcu | 56 to 77 | 21 | 74 | 29.73 | 0.0099 |
| 107 | bta-miR-382 | gaaguuguucgugguggauucg | uacuugaagaGAAGUUGUUCGUGGUGGAUUCGcuuuacuuaugacgaaucauucacggacaacacuuuuuucagua | 11 to 32 | 22 | 71 | 39.44 | 0.0099 |
| 108 | bta-miR-154b | agaggucuuccauggugcauucg | guacuugaagAGAGGUCUUCCAUGGUGCAUUCGcuuuauucuuugacgaaucauacaugguugaccuuuuuuuagguauca | 11 to 33 | 22 | 74 | 39.19 | 0.0099 |
| 109 | bta-miR-411c-5p | gguugaucagagaacauacauu | gggaguggauGGUUGAUCAGAGAACAUACAUUuugucaaugauguaugucaacugauccacagucccucccuau | 11 to 32 | 22 | 80 | 43.75 | 0.0099 |
| 110 | bta-miR-409b | gggguucaccgagcaacauuc | ugauaccgaaaaGGGGUUCACCGAGCAACAUUCgucguccagaugcaaaguugcucggguaaccucuccccgcguacca | 13 to 33 | 22 | 58 | 53.45 | 0.0099 |
| 111 | bta-miR-193b | aacuggcccacaaagucccgcuuu | guggucccagaaucgggguuuugagggcgagaugaguuuauguuuuauccAACUGGCCCACAAAGUCCCGCUUUuggggucau | 51 to 74 | 22 | 75 | 50.67 | 0.0099 |
| 112 | bta-miR-25 | cauugcacuugucucggucuga | ggccaguguugagaggcggagacuugggcaauugcuggacgcugccccgggCAUUGCACUUGUCUCGGUCUGAcagugccggcc | 52 to 73 | 22 | 93 | 62.37 | 0.0495 |
| 113 | bta-miR-486 | uccuguacugagcugccccgag | gccagcuuggaccugcguccucccugacgggUCCUGUACUGAGCUGCCCCGAGgcccuucgcugugcccagcucgggucagcucaguaccgggcgcgucggggugggagucggccggaagcagg | 32 to 52 | 22 | 78 | 70.51 | 0.0099 |
| 114 | bta-miR-197 | uucaccaccuucuccacccagc | ggggcugugccggguagagagggcagugggagguaagagcucuucacccUUCACCACCUUCUCCACCCAGCagggccagca | 50 to 71 | 22 | 75 | 64.00 | 0.0099 |
| 115 | bta-miR-30e-5p | uguaaacauccuugacuggaagcu | gggcagucuuugcuacUGUAAACAUCCUUGACUGGAAGCUguaaggcguugcaaggagcuuucagucggauguuuacagcggcaggcugcca | 17 to 40 | 22 | 76 | 48.68 | 0.0099 |
| 116 | bta-miR-331-3p | gccccugggccuauccuagaa | gaguuugguuuuguuuggguuuguucuagguauggucccagggaucccagaucaaaccagGCCCCUGGGCCUAUCCUAGAAccaaccuaa | 61 to 81 | 21 | 74 | 54.05 | 0.0099 |
| 117 | bta-miR-200c | uaauacugccggguaaugaugga | cgucuuacccagcaguguuugggugcugguugggagucucUAAUACUGCCGGGUAAUGAUGGAgg | 41 to 63 | 22 | 77 | 61.04 | 0.0099 |
| 118 | bta-let-7b | ugagguaguagguugugugguu | cggggUGAGGUAGUAGGUUGUGUGGUUucaggguagugauguugcccccucggaagauaacuauacaaccuacugccuucc | 6 to 27 | 22 | 84 | 52.38 | 0.0099 |
| 119 | bta-miR-181c | aacauucaaccugucggugaguuu | uugccaaggguuugggggAACAUUCAACCUGUCGGUGAGUUUgggcagcucaggcaaaccaucgaccguugaguggaccccgaggccuggaacugcc | 19 to 42 | 22 | 67 | 58.21 | 0.0099 |
| 120 | bta-miR-95 | uucaacggguauuuauugagca | aacacagcgggcgcucaauaaauguuuguugaauugagaugcgcuaaaUUCAACGGGUAUUUAUUGAGCAcccacucugug | 49 to 70 | 22 | 65 | 41.54 | 0.0099 |
| 121 | bta-miR-23a | aucacauugccagggauuucca | ggccggcugggguuccuggggaugggauuugcugccugucacaaAUCACAUUGCCAGGGAUUUCCAaccgacc | 45 to 66 | 22 | 56 | 53.57 | 0.0099 |
| 122 | bta-miR-145 | guccaguuuucccaggaaucccu | caccuuguccucacgGUCCAGUUUUCCCAGGAAUCCCUuagaugcuaagauggggauuccuggaaauacuguucuugaggucaugguu | 16 to 38 | 23 | 72 | 48.61 | 0.0099 |
| 123 | bta-miR-146a | ugagaacugaauuccauagguugu | cccauguguauccucagcuuUGAGAACUGAAUUCCAUAGGUUGUgucagugucagaccugugaaguuuaguucuuuagcugggauaucucuaucauccu | 21 to 44 | 22 | 67 | 41.79 | 0.0099 |
| 124 | bta-miR-31 | aggcaagaugcuggcauagcu | uccuguaacuuggaacuggagaggAGGCAAGAUGCUGGCAUAGCUguugaacugcgaaccugcuaugccaacauauugccaucucucuuguccg | 25 to 45 | 21 | 71 | 50.70 | 0.0099 |
| 125 | bta-miR-2468 | auaggaacauggaagauuguca | gauuggcAUAGGAACAUGGAAGAUUGUCAgucaucaucuauuucugccaauuuuccauguuccugugccaguc | 8 to 29 | 22 | 73 | 42.47 | 0.0099 |
| 126 | bta-miR-491 | aguggggaacccuuccaugagg | uugacuuagcuggguAGUGGGGAACCCUUCCAUGAGGaguagaacacuccuuaugcaagauucccuucuaccugacuggguugg | 16 to 37 | 22 | 62 | 50.00 | 0.0099 |
| 127 | bta-miR-23b-3p | aucacauugccagggauuaccac | ggguuccuggcaugcugauuugugacuuaagauuaaaAUCACAUUGCCAGGGAUUACCAC | 38 to 60 | 21 | 68 | 44.12 | 0.0099 |
| 128 | bta-miR-455-5p | uaugugccuuuggacuacauc | ucccuggcgugagggUAUGUGCCUUUGGACUACAUCguggaagccagcaccaugcaguccaugggcauauacacuugccucaaggccua | 16 to 36 | 22 | 58 | 51.72 | 0.0099 |
| 129 | bta-miR-20b | caaagugcucacagugcaggua | aguacCAAAGUGCUCACAGUGCAGGUAguuuuggcagcgcucuacuguagugugggcacuuccaguacu | 6 to 27 | 23 | 69 | 50.72 | 0.0099 |
| 130 | bta-miR-18b | uaaggugcaucuagugcaguua | cuuguguUAAGGUGCAUCUAGUGCAGUUAgugaagcagcucagaaucuacugcccuaaaugcuccuucuggcaca | 8 to 29 | 23 | 77 | 48.05 | 0.0198 |
| 131 | bta-miR-424-5p | cagcagcaauucauguuuuga | ucguugacuccgaggggaugCAGCAGCAAUUCAUGUUUUGAagugcuuuaaacgguucaaaacgugaggcgcugcuauacccccuugcgaggaagu | 21 to 41 | 21 | 55 | 43.64 | 0.0099 |
| 132 | bta-miR-503-5p | uagcagcgggaacaguacug | agccgugcccUAGCAGCGGGAACAGUACUGcagugggcaauuggugaucuggaguauuguuucugcugcccgggcaagacugg | 11 to 30 | 23 | 71 | 56.34 | 0.0099 |
| 133 | bta-miR-545-3p | aucaacaaacauuuauugugug | cccagccuggcacauucguaggccucaguaaauguuuauuggaugaauaaaugaauggcucAUCAACAAACAUUUAUUGUGUGccugcuaacgugaucuccacagg | 62 to 83 | 22 | 71 | 36.62 | 0.0099 |
| 134 | bta-miR-362-3p | aacacaccuauucaaggauuc | cucgaauccuuggaaccuaggugugagugcuguucuagugcAACACACCUAUUCAAGGAUUCaaa | 42to 62 | 22 | 72 | 47.22 | 0.0099 |
| 135 | bta-miR-500 | uaauccuugcuaccugggugaga | gcucccccucucUAAUCCUUGCUACCUGGGUGAGAgugcuuucugaaugcaaugcaccugggcaaggauucugagagagggagc | 13 to 35 | 23 | 76 | 52.63 | 0.0099 |
| 136 | bta-miR-1468 | cuccguuugccuguuuugcuga | guugaagaggugggugguuuCUCCGUUUGCCUGUUUUGCUGAuguucauuugacuuauucucagcaaaauaagcaaauggaaaauucauccaucaac | 21 to 42 | 22 | 91 | 37.36 | 0.0099 |
| 137 | bta-miR-499 | uuaagacuugcagugauguuu | gggcgggcggccgUUAAGACUUGCAGUGAUGUUUaacuccucuccacgugaacaucacagcaagucugugcugcuucccguccccacgcugccugggcagggu | 14 to 34 | 21 | 68 | 51.47 | 0.0099 |
| 138 | bta-miR-142-3p | aguguuuccuacuuuauggaug | gacagugcagucacccauaaaguagaaagcacuacuaacagcacuggaggguguAGUGUUUCCUACUUUAUGGAUGaguguacugug | 55 to 76 | 22 | 73 | 43.84 | 0.0198 |
| 139 | bta-miR-152 | ucagugcaugacagaacuuggg | uguccucccggcccagguucugugauacacuccgacucgggcucuggagcagUCAGUGCAUGACAGAACUUGGGcccggacggacc | 53 to 74 | 21 | 67 | 59.70 | 0.0099 |
| 140 | bta-miR-493 | ugaaggucuacugugugccagg | cuggcccccagggccuuguacaugguaggcuuucauucauucguuugcacauucggUGAAGGUCUACUGUGUGCCAGGcccugugccag | 57 to 78 | 22 | 75 | 52.00 | 0.0099 |
| 141 | bta-miR-299 | ugguuuaccgucccacauacau | aagaaaUGGUUUACCGUCCCACAUACAUucugaauauguaugugggacgguaaaccgcuucuu | 7 to 28 | 21 | 63 | 41.27 | 0.0099 |
| 142 | bta-miR-3957 | cucggagagugcagcugugg | uuggugggagCUCGGAGAGUGCAGCUGUGGGugucagggucaggcacacgcacagcaccucacugagcuccuuccaa | 11 to 30 | 22 | 75 | 62.67 | 0.0099 |
| 143 | bta-miR-92b | uauugcacucgucccggccucc | cgggccccgggcgggcgggagggacgggacgcggugcaguguuguucuuuccccugccaaUAUUGCACUCGUCCCGGCCUCCggcccccucggccc | 61 to 82 | 21 | 75 | 68.00 | 0.0099 |
| 144 | bta-miR-196b | uagguaguuuccuguuguuggga | aacuggucggugauuUAGGUAGUUUCCUGUUGUUGGGAuccaccuuucucucgacagcacgacacugccuucauuacuucaguug | 16 to 38 | 21 | 70 | 47.14 | 0.0099 |
| 145 | bta-miR-1271 | cuuggcaccuaguaaguacuca | cacccagaucagugCUUGGCACCUAGUAAGUACUCAguauauacuuguugagugccugcuaugugccaggcauugugcugagggcu | 15 to 36 | 22 | 66 | 46.97 | 0.0099 |
| 146 | bta-miR-532 | caugccuugaguguaggaccgu | gacuugcuuucucucuuaCAUGCCUUGAGUGUAGGACCGUuggcaucuuaauuacccucccacacccaaggcuugcaggagagcca | 19 to 40 | 22 | 73 | 52.05 | 0.0099 |
| 147 | bta-miR-188 | caucccuugcaugguggagggu | ugcucccucucucaCAUCCCUUGCAUGGUGGAGGGUgagcuuucugaaaaccccucccacaugcaggguuugcaggauggugagcc | 15 to 36 | 22 | 58 | 55.17 | 0.0198 |
| 148 | bta-miR-222 | agcuacaucuggcuacugggu | gcugcuggaauguguagguacccucaauggcucaguagccaguguagauccugucuuuuguaaucaguAGCUACAUCUGGCUACUGGGUcucugauggcaucuucuaccu | 69 to 89 | 21 | 62 | 48.39 | 0.0099 |
| 149 | bta-miR-425-5p | augacacgaucacucccguuga | gaaagcgcuuuggaAUGACACGAUCACUCCCGUUGAgugggcacccaagaagccaucgggaaugucguguccgcccagugcucuuuc | 15 to 36 | 23 | 67 | 58.21 | 0.0198 |
| 150 | bta-miR-27b | uucacaguggcuaaguucugc | accucucugacgaggugcagagcuuagcugauuggugaacagugacugguuuccgcuuugUUCACAGUGGCUAAGUUCUGCaccugaagagaaggug | 61 to 81 | 22 | 73 | 49.32 | 0.0099 |
| 151 | bta-miR-10b | uacccuguagaaccgaauuugug | cagugacguugucuauauaUACCCUGUAGAACCGAAUUUGUGugguauccauguagucacagauucgauucuaggggaauauauggucgaugcaaaaac | 20 to 43 | 22 | 74 | 39.19 | 0.0099 |
| 152 | bta-miR-107 | agcagcauuguacagggcuauc | cucucugcuuucagcuucuuuacaguguugccuuguggcauggaguucaAGCAGCAUUGUACAGGGCUAUCaaagcacaga | 50 to 71 | 21 | 70 | 47.14 | 0.0198 |
| 153 | bta-miR-192 | cugaccuaugaauugacagccag | agaccgagugcacagggcuCUGACCUAUGAAUUGACAGCCAGugcucuuguguccccucuggcugccaauuccauaggucacagguauguucgccucaaugccagc | 20 to 42 | 21 | 59 | 52.54 | 0.0099 |
| 154 | bta-miR-33a | gugcauuguaguugcauugca | cugcgGUGCAUUGUAGUUGCAUUGCAuguucuggcgguacccgugcaauguuuccacagugcaucacag | 6 to 26 | 21 | 69 | 52.17 | 0.0099 |
| 155 | bta-miR-27a-5p | agggcuuagcugcuugugagca | uggccuggggagcAGGGCUUAGCUGCUUGUGAGCAgguccacaucaaaucguguucacaguggcuaaguuccgccccc | 14 to 35 | 22 | 73 | 58.90 | 0.0099 |
| 156 | bta-miR-136 | acuccauuuguuuugaugaugga | uuggaugagcccucggaggACUCCAUUUGUUUUGAUGAUGGAuucuuacgcuccaucaucgucucaaaugagucuucagaggguuccaucau | 20 to 42 | 22 | 62 | 41.94 | 0.0099 |
| 157 | bta-miR-127 | ucggauccgucugagcuuggcu | ugaucacugucuccagccugcugaagcucagagggcucugauucagaaagaucaUCGGAUCCGUCUGAGCUUGGCUggucggaagucuccucauc | 55 to 76 | 22 | 72 | 55.56 | 0.0099 |
| 158 | bta-miR-1185 | agaggauacccuuuguauguu | uuugguacuugaagAGAGGAUACCCUUUGUAUGUUcacuuuauuaauggcgaauauacagagggagacucuuauuugcguaucaaa | 15 to 35 | 22 | 59 | 38.98 | 0.0099 |
| 159 | bta-miR-190b | ugauauguuugauauuggguu | ugcuucugugUGAUAUGUUUGAUAUUGGGUUguuuaauuaggaaccaacuaaaugucaaacauauucuuacagcaguag | 11 to 31 | 22 | 68 | 30.88 | 0.0099 |
| 160 | bta-miR-17-5p | caaagugcuuacagugcagguagu | gucagaauaauguCAAAGUGCUUACAGUGCAGGUAGUgauaugugcaucuacugcagugaaggcacuuguagcauuauggugac | 14 to 37 | 23 | 72 | 40.28 | 0.0099 |

* The bubaline miRNAs have been named according to the homology with the already reported taurine miRNAs in the miRBase (build 14)

** Pval: Probability value of the sequence being an miRNA
