# Supplementary material for: Identification of the MicroRNA Repertoire in TLR-Ligand Challenged Bubaline PBMCs as a Model of Bacterial and Viral Infection
Source: PLoS One. 2016 Jun 3;11(6):e0156598. doi: 10.1371/journal.pone.0156598 (PMC4892552; doi:10.1371/journal.pone.0156598)
Supplement: S3 Table — (DOCX) [file pone.0156598.s003.docx]

**S3Table. List of the novel miRNAs (with respect to taurine miRNAs) identified in the bubaline PBMCs treated with TLR ligands vis-à-vis the respective control groups**

| **SN** | **Arbitrary Id** | **Sequence**  **(5’-3’)** | **Hairpin Sequence**  **(5’-3’)** | **Position** | **sRNA Length** | **Hairpin Length** | **Hairpin GC** | **P value** |
| --- | --- | --- | --- | --- | --- | --- | --- | --- |
| 1 | bta-miR-11001 | gugaaaugauggcaaucaucuu | ccuuaaaGUGAAAUGAUGGCAAUCAUCUUucgggacugaccugaaaugaagagaauacucauugcugaucacuuuuauuguuuggg | 8 to 29 | 22 | 86 | 38.37 | 0.0099 |
| 2 | bta-miR-11002 | ggagagaacguaaucugaguggu | ccguuagccauugaugaucguucuucuuuuccuuuuaggagaugaagagGGAGAGAACGUAAUCUGAGUGGUuucgg | 50 to 72 | 23 | 77 | 44.16 | 0.0099 |
| 3 | bta-miR-11003 | gauguccagccacaauucucg | gccacaGAUGUCCAGCCACAAUUCUCGguuggccgcagacucguacaagaauugcguuuggacaaucaguggc | 7 to 27 | 21 | 73 | 53.42 | 0.0297 |
| 4 | bta-miR-11004 | gaagagccagcucauuagaaag | ucagggucuuuucuaaugagucagcucuuugcaucagguggccaaaguGAAGAGCCAGCUCAUUAGAAAGgacucuga | 49 to 70 | 22 | 78 | 46.15 | 0.0099 |
| 5 | bta-miR-11005 | uccacaucccucacaguuuggug | cuccgggUCCACAUCCCUCACAGUUUGGUGcuggaaugaaaaccccaggcuguuggaauguugacccaggg | 8 to 30 | 23 | 71 | 56.34 | 0.0099 |
| 6 | bta-miR-11006 | uugcagugaugacuugagucu | ugcuggcuUUGCAGUGAUGACUUGAGUCUgucaauccccugagugaguuuaaucacugaugucuccaugucucugagcaaagccucugagua | 9 to 29 | 21 | 92 | 46.74 | 0.0792 |
| 7 | bta-miR-11007 | cuucagugaugacacgaugacga | uugguuuguccuugucucagccacugguguaccCUUCAGUGAUGACACGAUGACGAgucag | 34 to 56 | 23 | 61 | 50.82 | 0.0099 |
| 8 | bta-miR-11008 | gauuagaggcauuugucugaga | gguuccuuuugcagaguguccuguucauugauuGAUUAGAGGCAUUUGUCUGAGAaggggcc | 34 to 55 | 22 | 62 | 46.77 | 0.0099 |
| 9 | bta-miR-11009 | cuccuggggcccgcacucucg | ucggugcuggggagcggcccccgggcgggccucugcucuggcccCUCCUGGGGCCCGCACUCUCGcuccgg | 45 to 65 | 21 | 71 | 80.28 | 0.0198 |
| 10 | bta-miR-11010 | uauucauuuaucucccagccua | aggcuaggagaaaugauuggauagaaaauuuuauucUAUUCAUUUAUCUCCCAGCCU | 37 to 57 | 22 | 57 | 33.33 | 0.0099 |
| 11 | bta-miR-11011 | gucggguugcuugggaaugca | gguagcggcccccggcgcgccgggcccgggucuucccggaGUCGGGUUGCUUGGGAAUGCAgcc | 41 to 61 | 21 | 64 | 76.56 | 0.0594 |
| 12 | bta-miR-11013 | ggcgcucccuccccgcggggg | gccGGCGCUCCCUCCCCGCGGGGGggccgcuccccgcgaggggggcguuccccgcggggcgcgcgccggu | 4 to 24 | 21 | 70 | 90.00 | 0.0099 |
| 13 | bta-miR-11018 | gagcccagcgccgaauccccg | gggaaGAGCCCAGCGCCGAAUCCCCGccccgcgguggggcgugggacauguggcguacggaagacccacccccggcgccgcucguguggggccc | 6 to 26 | 21 | 94 | 76.60 | 0.0990 |
| 14 | bta-miR-11024 | acccugauugcuccuaucugauu | cagaauagaacaugaugauuggagcucgcaugauucgugauuaacggcucugcguaaucaggacuugcaacACCCUGAUUGCUCCUAUCUGAUUcuuccug | 72 to 94 | 23 | 101 | 45.54 | 0.0198 |
| 15 | bta-miR-11025 | aaaacuggaacgaacuuuugg | guugaacaaaaaguucguccagauuuuuccauaagauguuacagaAAAACUGGAACGAACUUUUGGgccaac | 46 to 66 | 21 | 72 | 37.50 | 0.0099 |
| 16 | bta-miR-11026 | uacccuguagaaccgaauuug | cuauauaUACCCUGUAGAACCGAAUUUGugugguauccauguagucacagauucgauucuaggggaauauaugg | 8 to 28 | 21 | 74 | 39.19 | 0.0099 |
| 17 | bta-miR-11027 | aaaaccugaacgagccuuuugg | uggguuggugaaaagguucauuuggguuuuucuguaagauguuacagaAAAACCUGAACGAGCCUUUUGGccaaccca | 49 to 70 | 22 | 78 | 42.31 | 0.0099 |
| 18 | bta-miR-11028 | gcaugggugguucagugguaga | uaccacagugaacuccaugccuuccugagcaagcccuaccuaauaaggccaaucaggagucuagagcagguGCAUGGGUGGUUCAGUGGUA | 72 to 91 | 22 | 91 | 51.65 | 0.0297 |
| 19 | bta-miR-11029 | gcguuggugguauaguggugagc | ucuuucucaucacuuaaacucagcaugugcugguGCGUUGGUGGUAUAGUGGUGAGCguaga | 35 to 57 | 23 | 62 | 46.77 | 0.0594 |
| 20 | bta-miR-11030 | uucagcuguuuugugagacauuc | gagaagaagagauaauucacggagcuagcguuguugccuucugucuucacagaggccuaUUCAGCUGUUUUGUGAGACAUUCuguuuc | 60 to 82 | 23 | 88 | 44.32 | 0.0297 |
| 21 | bta-miR-11031 | gucuucucacugagcuccuuucu | UCUUCUCACUGAGCUCCUUUCUgucaaucagugacaguugaggauucgcacgagaaga | 1 to 22 | 23 | 58 | 46.55 | 0.0198 |
| 22 | bta-miR-11032 | gaucgaugaugacacccucaca | cucgGAUCGAUGAUGACACCCUCACAugcauuccuuggaaaaucugaacaaaaugagugagaacucacuacugucuccucaucgaaacugag | 5 to 26 | 22 | 92 | 44.57 | 0.0198 |
| 23 | bta-miR-11033 | acuccauuuguuuugaugaug | gaggACUCCAUUUGUUUUGAUGAUGgauucuuacgcuccaucaucgucucaaaugagucuuc | 5 to 25 | 21 | 62 | 41.94 | 0.0099 |
| 24 | bta-miR-11034 | aacugucaaucgaugggacagag | gauaucuuugcccugggacugugucaguggcAACUGUCAAUCGAUGGGACAGAGaguauuc | 32 to 54 | 23 | 61 | 49.18 | 0.0792 |
| 25 | bta-miR-11035 | auugaggucuaucccgauggggc | UGAGGUCUAUCCCGAUGGGGCuuuuucuauaaaccuacaucguuggaaacgccuca | 1 to 21 | 23 | 56 | 46.43 | 0.0198 |
| 26 | bta-miR-11036 | gaaaaguucguuuggguuuuc | gguuggccGAAAAGUUCGUUUGGGUUUUCcauaacaucuuccagaaaaaccugaacaaauuuucuggccaacc | 9 to 29 | 21 | 73 | 42.47 | 0.0099 |
| 27 | bta-miR-11037 | ugaccuaugaauugacagccagu | cucUGACCUAUGAAUUGACAGCCAGUgcucuuguguccccucuggcugccaauuccauaggucacag | 4 to 26 | 23 | 67 | 52.24 | 0.0099 |
| 28 | bta-miR-11038 | ucacuucagccuuuucuuucuag | UCACUUCAGCCUUUUCUUUCUAGcuguacuaaaugagcagaugaaggaggcugaagga | 1 to 23 | 23 | 58 | 43.10 | 0.0198 |
| 29 | bta-miR-11039 | ggcugguccgaugguagugggu | GGCUGGUCCGAUGGUAGUGGGUuaccagaacuuauuaacguuagugucacuaaaguugguauacaaccccccacugcuaaauuugacuggcu | 1 to 22 | 22 | 92 | 45.65 | 0.0396 |
| 30 | bta-miR-11040 | cccccacugcuaaauuugacugg | ggcugguccgaugguaguggguuaccagaacuuauuaacguuagugucacuaaaguugguauacaacCCCCCACUGCUAAAUUUGACUGGcu | 68 to 90 | 23 | 92 | 45.65 | 0.0099 |
| 31 | bta-miR-11041 | aaacucgaaugaacucuuugg | uggguuagccaaaaagaucauuuggguuuuucugaugauguuauggaAAACUCGAAUGAACUCUUUGGcugacuca | 48 to 68 | 21 | 76 | 38.16 | 0.0099 |
| 32 | bta-miR-11042 | cagcaugugaaaucuuugguug | acuugcagcaacuaaaaauuccacaugcucucuaacaaaguuaaucggcuguaCAGCAUGUGAAAUCUUUGGUUGaugcaugu | 54 to 75 | 22 | 83 | 38.55 | 0.0099 |
| 33 | bta-miR-11045 | gggaagugaugauacuuauga | cGGGAAGUGAUGAUACUUAUGAcuccugacagugcgcugauuucucaccguauucguacuggcugaucccgaguguccccuuccug | 2 to 22 | 21 | 86 | 52.33 | 0.0198 |
| 34 | bta-miR-11046 | gcguuggugguauagugguuagc | gcuaauacuuuuacuaagggcauugugagaaacccucacuuacugggGCGUUGGUGGUAUAGUGGUUAGC | 48 to 70 | 23 | 70 | 45.71 | 0.0792 |
| 35 | bta-miR-11049 | uccuggugaugacagaugacau | ggaggaUCCUGGUGAUGACAGAUGACAUugucagccaauccccacaugggagugaggacauguccugcaauucugaagggauucucu | 7 to 28 | 22 | 87 | 50.57 | 0.0693 |
| 36 | bta-miR-11050 | uccuagucugggugcaaacaguu | uguuuugcaccucugagaguggaguaacuccuccuguggaguuggUCCUAGUCUGGGUGCAAACA | 46 to 65 | 23 | 65 | 50.77 | 0.0099 |
| 37 | bta-miR-11051 | uauaccaggaugccagcauaguu | gauagcugguuggcauucugggccugguucaugccuacucuguuauuaacUAUACCAGGAUGCCAGCAUAGUUguu | 51 to 73 | 23 | 76 | 46.05 | 0.0099 |
| 38 | bta-miR-11052 | uauugcacaugacuaaguugc | guggaggUAUUGCACAUGACUAAGUUGCauguugucacggccucagugcaauuuaguaugugugauauuuucac | 8 to 28 | 21 | 74 | 41.89 | 0.0099 |
| 39 | bta-miR-11054 | gcggcccgcggcggcgggcgcg | ucGCGGCCCGCGGCGGCGGGCGCGucggucccccccgccgggugcgcccccggggccgcgg | 3 to 24 | 22 | 61 | 93.44 | 0.0693 |
| 40 | bta-miR-11055 | agcuacaucuggcuacugggucu | ggcucaguagccaguguagauccugucuuuuguaaucaguAGCUACAUCUGGCUACUGGGUC | 41 to 62 | 23 | 62 | 48.39 | 0.0099 |
| 41 | bta-miR-11056 | gaaaaguucguuuggguuuucu | guuggccGAAAAGUUCGUUUGGGUUUUCUauaccacaguaagaaaaaaaccaaacgauuuuuuuggccaac | 8 to 29 | 22 | 71 | 38.03 | 0.0099 |
| 42 | bta-miR-12001 | ugcacgaggccccggguucaa | ggggguguagcucagugguagagcgcgugcuuagcaUGCACGAGGCCCCGGGUUCAAucccc | 37 to 57 | 21 | 62 | 64.52 | 0.0198 |
| 43 | bta-miR-12002 | gaggauuuagcuuaauuaaagug | guuaacuaaaguuucauggggguagaucccaccagucuaguGAGGAUUUAGCUUAAUUAAAGUGguugau | 42 to 64 | 23 | 70 | 38.57 | 0.0396 |
| 44 | bta-miR-12003 | caaccauaggcagucacauaca | gaccugugugacuguuccugguauugauugcauuuguuggucguacagugCAACCAUAGGCAGUCACAUACAuc | 51 to 72 | 22 | 74 | 45.95 | 0.0198 |
| 45 | bta-miR-12004 | auccagcaguugucagcuauc | ucAUCCAGCAGUUGUCAGCUAUCcaggcuagcgugaugccucagaugguguuacacuguuggaaga | 3 to 23 | 21 | 66 | 50.00 | 0.0198 |
| 46 | bta-miR-12005 | uucaauggcugaggugagguacc | ccuggcggaugccuccuuagccggagcuuggaauagacucacggccggcgaagugagUUCAAUGGCUGAGGUGAGGUACCccgccugg | 57 to 80 | 23 | 88 | 62.50 | 0.0297 |
| 47 | bta-miR-12006 | ucaggguccgugagcuccucg | CAGGGUCCGUGAGCUCCUCGgcguacucggagaacauucgccggaacuccggguccug | 1 to 21 | 21 | 58 | 65.52 | 0.0198 |
| 48 | bta-miR-12007 | uguaaucccagcuacucggga | gcgcgccUGUAAUCCCAGCUACUCGGGAggcugaggcuggaggaucgcuugagcucaggaguucugggcugcagugcgc | 8 to 28 | 21 | 79 | 64.56 | 0.0792 |
| 49 | bta-miR-12008 | uguagucccagcuacucggga | gcgcgccUGUAGUCCCAGCUACUCGGGAggcugaggcuggaggaucgcuugagcucaggaguucugggcugcagugcgc | 8 to 28 | 21 | 79 | 65.82 | 0.0198 |
| 50 | bta-miR-12009 | uaugcaucuaugugugugugu | acacgcacacauauauauguauauauauacacauacacacauagauguauauauguguguauguguauauaUAUGCAUCUAUGUGUGUGUGUgu | 72 to 92 | 21 | 94 | 29.79 | 0.0099 |
| 51 | bta-miR-12010 | augcugacauauuuacuagag | cuuccucAUGCUGACAUAUUUACUAGAGgguaaaauuaauaaccuucuaguaagaguggcagucgaagggaag | 8 to 28 | 21 | 73 | 39.73 | 0.0099 |
| 52 | bta-miR-12011 | aacgaagacuucagggcagcu | cAACGAAGACUUCAGGGCAGCUggaaagucaggaucugcucuugagccuucagug | 2 to 22 | 21 | 55 | 52.73 | 0.0693 |
| 53 | bta-miR-12012 | gaggacucagcaguaggcucu | uggguaGAGGACUCAGCAGUAGGCUCUgugagagacccucaggaguaggcuguggagaccaagacuca | 7 to 27 | 21 | 68 | 55.88 | 0.0297 |
| 54 | bta-miR-12013 | ggagagaacguaaucugagugg | ccguuagccauugaugaucguucuucuuuuccuuuuaggagaugaagagGGAGAGAACGUAAUCUGAGUGGuuucgg | 50 to 71 | 22 | 77 | 44.16 | 0.0099 |
| 55 | bta-miR-12015 | ucucagugaugcgauuccaau | ucgUCUCAGUGAUGCGAUUCCAAUagauccuucugacccuccacuguggacucaauagcagggagaugaagaggaacgugacugagagacga | 4 to 24 | 21 | 92 | 50.00 | 0.0693 |
| 56 | bta-miR-12017 | gggcagagaggaagcggcuau | agguguggccgcccugcugcgccagugccguugGGGCAGAGAGGAAGCGGCUAUuucu | 34 to 54 | 21 | 58 | 65.52 | 0.0396 |
| 57 | bta-miR-12018 | ggcagagaggaaagaggcucg | ucgugaucuuccccgcacugccccuagaggcgcugcaggcguggcugccgugucacgucugcgucaugagguGGCAGAGAGGAAAGAGGCUCGg | 73 to 93 | 21 | 94 | 64.89 | 0.0198 |
| 58 | bta-miR-12019 | guccggggaauggauccagcgu | acugaacacacuggauucauuucucugaaaguggauuuuguggucuGUCCGGGGAAUGGAUCCAGCGUgaucggu | 47 to 68 | 22 | 75 | 48.00 | 0.0099 |
| 59 | bta-miR-12022 | cccccgaguguuacagccccc | ggugggggcgcgguacugugggggcaggccaaCCCCCGAGUGUUACAGCCCCCagc | 33 to 53 | 21 | 56 | 73.21 | 0.0396 |
| 60 | bta-miR-12023 | uugcagugaugacuugagucug | ugcuggcuUUGCAGUGAUGACUUGAGUCUGucaauccccugagugaguuuaaucacugaugucuccaugucucugagcaaagccucugagua | 9 to 30 | 22 | 92 | 46.74 | 0.0891 |
| 61 | bta-miR-12024 | ugccaagcccacguucaaaggcu | gccugcUGCCAAGCCCACGUUCAAAGGCUguuucuucaaaguuaauggugccgccuuugagcugggaagggaagcgggc | 7 to 29 | 23 | 79 | 56.96 | 0.0099 |
| 62 | bta-miR-12025 | aaacccgaauuaacuuuuugg | gguuggccagaaguucauuugggucuuuccguaacaucugauggaAAACCCGAAUUAACUUUUUGGccaacu | 46 to 66 | 21 | 72 | 43.06 | 0.0099 |
| 63 | bta-miR-12026 | gucgaggcuagagucacgcuugg | UCGAGGCUAGAGUCACGCUUGGguauccacuguugccuuagugugcuagaguccucga | 1 to 22 | 23 | 58 | 53.45 | 0.0099 |
| 64 | bta-miR-12027 | cuucagugaugacacgaugacg | uugguuuguccuugucucagccacugguguaccCUUCAGUGAUGACACGAUGACGagucag | 34 to 55 | 22 | 61 | 50.82 | 0.0198 |
| 65 | bta-miR-12028 | aaaaccagaacgaacuuugugu | guuggccaaaaaguucauucagauuuuucuaaaacagcuuacggaAAAACCAGAACGAACUUUGUGUccaac | 46 to 67 | 22 | 72 | 37.50 | 0.0099 |
| 66 | bta-miR-12029 | ggacaucugagauuguugaugaa | gGGACAUCUGAGAUUGUUGAUGAAugccaacggcucugauggcggcguaacccgacucaccggaugggucuuuguuaauaaccugggaaugaacc | 2 to 24 | 23 | 95 | 51.58 | 0.0495 |
| 67 | bta-miR-12030 | gcggggguggggggggcggcg | guuuuccuccccuacacggucaauauaaaaaugauuagugagaaauucuacaugcuuuguggcgggGCGGGGGUGGGGGGGGCGGC | 67 to 86 | 21 | 86 | 53.49 | 0.0891 |
| 68 | bta-miR-12031 | agcacagcuaccccgguacacgg | gggcAGCACAGCUACCCCGGUACACGGgugcuuucugggggugggcuggcugugucuccc | 5 to 27 | 23 | 60 | 68.33 | 0.0990 |
| 69 | bta-miR-12032 | gaaaagagcaccggcugagacgc | ggcuggcgucggugauguucugcaguuaccuacauuguuugaucccuguGAAAAGAGCACCGGCUGAGACGCcugcc | 50 to 72 | 23 | 77 | 55.84 | 0.0792 |
| 70 | bta-miR-12034 | ccccgaguguuacagccccccg | gccggcggggaagguggcucggggggguugccccgccgucgucacggcggcggguccgcccuucgCCCCGAGUGUUACAGCCCCCCGgc | 66 to 87 | 22 | 89 | 79.78 | 0.0099 |
| 71 | bta-miR-12043 | ccgaggugggaucccgaggcc | gcggCCGAGGUGGGAUCCCGAGGCCucucccaguccgccgagggcgcaccaccggccc | 5 to 25 | 21 | 58 | 79.31 | 0.0099 |
| 72 | bta-miR-12047 | ucccggggccgggagcggccgg | ggguUCCCGGGGCCGGGAGCGGCCGGggccccgguggcggggggaggucuuccccccgcggaggcccgggcacccggggggcc | 5 to 25 | 22 | 83 | 87.95 | 0.0792 |
| 73 | bta-miR-12048 | acucaaaacauggcggcacuu | ACUCAAAACAUGGCGGCACUUcccuucguaaaacggaagugcccccacaguuugagu | 1 to 21 | 21 | 57 | 50.88 | 0.0099 |
| 74 | bta-miR-12050 | ccaugaggaggagaugcagug | caaaaCCAUGAGGAGGAGAUGCAGUGuccucuccugagcgugaagcugguccuuggucuuguuucugcagcugcugacaccgucugagugguuuug | 6 to 26 | 21 | 96 | 53.13 | 0.0099 |
| 75 | bta-miR-12051 | uagcuuaucagacugauguug | ugucgggUAGCUUAUCAGACUGAUGUUGacuguugaaucucauggcaacagcagucgaugggcugucugaca | 8 to 28 | 21 | 72 | 48.61 | 0.0099 |
| 76 | bta-miR-12053 | auggauuuuggagcagggagu | uguaguaucuguucuuaucaguuuaauaucugauacguccucuauccgaggacaauauauuaaAUGGAUUUUGGAGCAGGGAGUgga | 64 to 84 | 21 | 87 | 36.78 | 0.0198 |
| 77 | bta-miR-12055 | acgcgcugccuuugagcccccg | GCGCUGCCUUUGAGCCCCCGccgcgccugcgcguggcgccgggggucaggcugcagcgc | 1 to 20 | 22 | 59 | 81.36 | 0.0198 |
| 78 | bta-miR-12058 | gcaugggugguucagugguagaa | uaccacagugaacuccaugccuuccugagcaagcccuaccuaauaaggccaaucaggagucuagagcagguGCAUGGGUGGUUCAGUGGUA | 72 to 91 | 23 | 91 | 51.65 | 0.0594 |
| 79 | bta-miR-12059 | uguccuugcuguuuggagaua | ucccagauacaauggacaauaugcuguuauaauuguguggcauUGUCCUUGCUGUUUGGAGA | 44 to 62 | 21 | 62 | 40.32 | 0.0099 |
| 80 | bta-miR-12061 | aggauucgcacgagaagaaga | ucuucucacugagcuccuuucugucaaucagugacaguugAGGAUUCGCACGAGAAGA | 41 to 58 | 21 | 58 | 46.55 | 0.0198 |
| 81 | bta-miR-12062 | gagcgugaugauuggguguucac | uGAGCGUGAUGAUUGGGUGUUCACgcuauugcgugauucgugcagaccuuguuacgacacuggcacauuacccaucugacgugaca | 2 to 24 | 23 | 86 | 50.00 | 0.0198 |
| 82 | bta-miR-12064 | cagaggggccuucucugcccgc | gcugguaagggaagccguccuucugcucugcccucuuagcuuCAGAGGGGCCUUCUCUGCCCGC | 43 to 64 | 22 | 64 | 62.50 | 0.0594 |
| 83 | bta-miR-12065 | caggagcgguuugcugccagcc | ggCAGGAGCGGUUUGCUGCCAGCCcgggccccaacucacuggccgccucccgcuccucc | 3 to 24 | 22 | 59 | 74.58 | 0.0198 |
| 84 | bta-miR-12068 | gaggugucaggauggccgagug | cGAGGUGUCAGGAUGGCCGAGUGgucuaaggcgccagacucaagcgaagcuuccccaccuug | 2 to 23 | 22 | 62 | 61.29 | 0.0297 |
| 85 | bta-miR-12073 | ucaauccccggcaccuccacca | gguUCAAUCCCCGGCACCUCCACCAguuuugagggggaugugcuugugaucaagcc | 4 to 25 | 22 | 56 | 57.14 | 0.0891 |
| 86 | bta-miR-12075 | uugcaugacucugagaguaag | aggaggcacuugcuuucagggaccugcaaguacugguccaucauUUGCAUGACUCUGAGAGUAAgugccuccu | 45 to 65 | 21 | 73 | 50.68 | 0.0099 |
| 87 | bta-miR-12076 | ggagaacugaauauaugcuagcu | GAGAACUGAAUAUAUGCUAGCUgucauucuuaccaucagcagcauauuuucagcacuu | 1 to 22 | 23 | 58 | 37.93 | 0.0099 |
| 88 | bta-miR-12079 | ugggagcgggcgggcgguccgcc | ggaucuUGGGAGCGGGCGGGCGGUCCGCCgcgaggcgagccaccgcccguccccgcccc | 7 to 29 | 23 | 59 | 83.05 | 0.0099 |
| 89 | bta-miR-12087 | ccaaaccaguugugccuguag | ucacaggcacggccaguuugagcauuuucacugaauugCCAAACCAGUUGUGCCUGUAGa | 39 to 59 | 21 | 60 | 48.33 | 0.0099 |
| 90 | bta-miR-12088 | cagcguuacacugcugggaga | uuccgugguucccagcaguagucagcugccgggacagccauuccugugaCAGCGUUACACUGCUGGGAGAggaa | 50 to 70 | 21 | 74 | 58.11 | 0.0099 |
| 91 | bta-miR-12092 | gagcaccugaaucuuucccauu | AGCACCUGAAUCUUUCCCAUUcucugcugcuucgugcugguguggggacagauggugcu | 1 to 21 | 22 | 59 | 54.24 | 0.0198 |
| 92 | bta-miR-12093 | uccaaugggguuuccccgcacag | gaaaUCCAAUGGGGUUUCCCCGCACAGguucgaauccuguucgugacgggcgcuuuuuuuccccuucagggugugugugaaggaaaccucuuggcuuc | 5 to 27 | 23 | 98 | 53.06 | 0.0495 |
| 93 | bta-miR-12096 | ggacuccggcaggacgcacagca | cugugguuuuucuguuccccugugcucaguucucaggGGACUCCGGCAGGACGCACAG | 38 to 58 | 23 | 58 | 58.62 | 0.0495 |
| 94 | bta-miR-12098 | cccccacugcuaaauuugacug | aggcugguccgaugguaguggguuaccagaacuuauuaacauuagugucacuaaaguugguauacaacCCCCCACUGCUAAAUUUGACUGgcuu | 68 to 90 | 22 | 94 | 43.62 | 0.0099 |
| 95 | bta-miR-12100 | cuggcaccugcagggauugcu | gcaggcuagugucugcaggucccaagcccucuguucaggaucCUGGCACCUGCAGGGAUUGCUGccugc | 43 to 63 | 21 | 69 | 62.32 | 0.0396 |
| 96 | bta-miR-12101 | ccuggugaugacagaugacauug | ggaggauCCUGGUGAUGACAGAUGACAUUGucagccaauccccacaugggagugaggacauguccugcaauucugaagggauucucu | 8 to 30 | 23 | 87 | 50.57 | 0.0990 |
| 97 | bta-miR-12105 | guccaguuuucccaggaaucc | ccucacgGUCCAGUUUUCCCAGGAAUCCcuuagaugcuaagauggggauuccuggaaauacuguucuugagg | 8 to 28 | 21 | 72 | 48.61 | 0.0099 |
| 98 | bta-miR-12106 | aguggggaacccuuccaugag | ggguAGUGGGGAACCCUUCCAUGAGgaguagaacacuccuuaugcaagauucccuucuaccu | 5 to 25 | 21 | 62 | 50.00 | 0.0099 |
| 99 | bta-miR-12107 | aagaguucauucagguuuuuug | uggccgAAGAGUUCAUUCAGGUUUUUUGguaagaucuuacagaaaaaccugaaugaacgcuuuagcca | 7 to 28 | 22 | 68 | 39.71 | 0.0099 |
| 100 | bta-miR-12110 | aaaacuggaaugaacuuuuugc | uggguugaccaauaguucauuuggauuuuuccauagcaucuuacggaAAAACUGGAAUGAACUUUUUGCcaaccca | 48 to 69 | 22 | 76 | 38.16 | 0.0099 |
| 101 | bta-miR-12112 | cgucaaccauccagcuguuug | aaaaguucCGUCAACCAUCCAGCUGUUUGaggugaugcaaacaaacaucugguugguugagagaauuuuu | 9 to 29 | 21 | 70 | 40.00 | 0.0099 |
| 102 | bta-miR-12113 | ugugacagauugauaacugaa | cagacaucucggggaucaucaugucacgagauaccacugugcacuUGUGACAGAUUGAUAACUGAAaggucug | 46 to 66 | 21 | 73 | 46.58 | 0.0099 |
| 103 | bta-miR-13001 | gaaaaguucguucggguuuuuc | uauuggguuggcuGAAAAGUUCGUUCGGGUUUUUCcauaagaacucaaauaaacuuuuuggccaacccagua | 14 to 35 | 22 | 72 | 38.89 | 0.0099 |
| 104 | bta-miR-13007 | uagcagcacguaaauauuggugu | UAGCAGCACGUAAAUAUUGGUGUuaagauucuaaaauuaucuccaguauuaacugugcugcug | 1 to 23 | 23 | 63 | 34.92 | 0.0099 |
| 105 | bta-miR-13008 | agagguguagaauaaguggga | caugAGAGGUGUAGAAUAAGUGGGAggcccccggcgcccccccgcccccguuucccgcgagggggcgggguguguccgacucuuagcgaccgcaug | 5 to 25 | 21 | 96 | 69.79 | 0.0396 |
| 106 | bta-miR-13011 | gagucggguugcuugggaaugca | gguagcggcccccggcgcgccgggcccgggucuucccgGAGUCGGGUUGCUUGGGAAUGCAgcc | 39 to 61 | 23 | 56 | 76.79 | 0.0297 |
| 107 | bta-miR-14020 | ugauauagacagcaggacggu | guugguUGAUAUAGACAGCAGGACGGUggccauggaagucggaauccgcuaaggaguguguaacaacucaccugccgaaucaacuagc | 7 to 27 | 21 | 88 | 51.14 | 0.0990 |
| 108 | bta-miR-13025 | uugcaugacucugagaguaagu | aaggaggcacuugcuuucagggaccugcaaguacugguccaucauUUGCAUGACUCUGAGAGUAAGUgccuccuu | 46 to 67 | 22 | 75 | 49.33 | 0.0099 |
| 109 | bta-miR-13027 | uucaccaccuucuccacccag | ggguagagagggcagugggagguaagagcucuucacccUUCACCACCUUCUCCACCC | 39 to 57 | 21 | 57 | 59.65 | 0.0198 |
| 110 | bta-miR-13036 | gggcgcgcgccgcggcuggacga | gucggucgggcuggggcgcgaagcggggcuGGGCGCGCGCCGCGGCUGGACGAggc | 31 to 53 | 23 | 56 | 83.93 | 0.0198 |
| 111 | bta-miR-13038 | ucaacaaacauuuauugugugcc | guaggccucaguaaauguuuauuggaugaauaaaugaauggcucaUCAACAAACAUUUAUUGUGUGCCugc | 46 to 68 | 23 | 71 | 36.62 | 0.0099 |
| 112 | bta-miR-14005 | aucucagguuugucagcccgc | ggugccuucaggacuguccaaccugagaauggugagcauccagggacaAUCUCAGGUUUGUCAGCCCGCaaggugcc | 49 to 69 | 21 | 77 | 57.14 | 0.0099 |
| 113 | bta-miR-14032 | gggaagugaugauacuuaugac | cGGGAAGUGAUGAUACUUAUGACuccugacagugcgcugauuucucaccguauucguacuggcugaucccgaguguccccuuccug | 2 to 23 | 22 | 86 | 52.33 | 0.0198 |
| 114 | bta-miR-14038 | uauugcacaugacuaaguugca | guggaggUAUUGCACAUGACUAAGUUGCAuguugucacggccucagugcaauuuaguaugugugauauuuucac | 8 to 29 | 22 | 74 | 41.89 | 0.0099 |
| 115 | bta-miR-15005 | uccacaucccucacaguuuggu | cuccgggUCCACAUCCCUCACAGUUUGGUgcuggaaugaaaaccccaggcuguuggaauguugacccaggg | 8 to 29 | 22 | 71 | 56.34 | 0.0099 |
| 116 | bta-miR-15008 | acaggcuaggagaaaugauugg | AGGCUAGGAGAAAUGAUUGGauagaaaauuuuauucuauucauuuaucucccagccu | 1 to 20 | 22 | 57 | 33.33 | 0.0099 |
| 117 | bta-miR-16024 | aaaccguuaccauuacugagu | uugggguggcgaggAAACCGUUACCAUUACUGAGUuuaguaaugguaacgguucucuugcugcacccag | 15 to 35 | 21 | 69 | 49.28 | 0.0099 |
| 118 | bta-miR-16042 | agcagaguggcgcagcggaagc | guuAGCAGAGUGGCGCAGCGGAAGCgugcugggcccauaacccagaggucgauggaucgaaaccauccucugcuauuugcggugucuuuuuguugau | 4 to 25 | 22 | 97 | 53.61 | 0.0297 |
| 119 | bta-miR-15042 | ccucauguggaaucuuuaguug | ugcggcaacuaaagauuccacaugcuguacaacaaagugaauccgauguaCCUCAUGUGGAAUCUUUAGUUGuggca | 50 to 72 | 22 | 77 | 42.86 | 0.0099 |
| 120 | bta-miR-15056 | uaauccuugcuaccugggugag | cccucucUAAUCCUUGCUACCUGGGUGAGagugcuuucugaaugcaaugcaccugggcaaggauucugagagaggg | 8 to 29 | 22 | 76 | 52.63 | 0.0099 |
| 121 | bta-miR-16002 | gcacgaggccccggguucaaucc | uguaggggguguagcucagugguagagcgcgugcuuagcauGCACGAGGCCCCGGGUUCAAUCCccggca | 42 to 64 | 23 | 70 | 62.86 | 0.0099 |
| 122 | bta-miR-16004 | gcagagaggaaagaggcucgg | cgugaucuuccccgcacugccccuagaggcgcugcaggcguggcugccgugucacgucugcgucaugaggugGCAGAGAGGAAAGAGGCUCG | 73 to 92 | 21 | 92 | 65.22 | 0.0396 |
| 123 | bta-miR-16005 | uagcagcacguaaauauuggu | ugccuUAGCAGCACGUAAAUAUUGGUguuaagauucuaaaauuaucuccaguauuaacugugcugcugaagua | 6 to 26 | 21 | 73 | 35.62 | 0.0099 |
| 124 | bta-miR-16008 | gcggguaaacggcgggaguaac | gugauuucugccuauugcucugaauguuaaagugaagaaauucaaugaagcGCGGGUAAACGGCGGGAGUAAC | 52 to 73 | 22 | 73 | 43.84 | 0.0198 |
| 125 | bta-miR-16011 | gaaaagagcaccggcugagacg | ggcuggcgucggugauguucugcaguuaccuacauuguuugaucccuguGAAAAGAGCACCGGCUGAGACGccugcc | 50 to 71 | 22 | 77 | 55.84 | 0.0792 |
| 126 | bta-miR-16039 | guaugaggccccggguucgau | ggggauguagcucagugguagagcgcaugcuucgcauGUAUGAGGCCCCGGGUUCGAUcccc | 38 to 58 | 21 | 62 | 61.29 | 0.0891 |
| 127 | bta-miR-16044 | auugaggucuaucccgauggg | UGAGGUCUAUCCCGAUGGGgcuuuuucuauaaaccuacaucguuggaaacgccuca | 1 to 19 | 21 | 56 | 46.43 | 0.0099 |
| 128 | bta-miR-16045 | ggaccagagcacccuagaagc | gaggcuuugguuugcacgguccauucuaaucccugccggucagccuguggccugccaggcuucgcuuguGGACCAGAGCACCCUAGAAGCcuu | 70 to 90 | 21 | 93 | 59.14 | 0.0297 |
| 129 | bta-miR-16058 | ugagaacugaauuccauaggu | ucagcuuUGAGAACUGAAUUCCAUAGGUugugucagugucagaccugugaaguuuaguucuuuagcugg | 8 to 28 | 21 | 69 | 42.03 | 0.0099 |
| 130 | bta-miR-16059 | ccaauuuuccauguuccugugc | gauuggcauaggaacauggaagauugucagucaucaucuauuucugCCAAUUUUCCAUGUUCCUGUGCcaguc | 47 to 68 | 22 | 73 | 42.47 | 0.0099 |
