# Supplementary material for: Identification of the MicroRNA Repertoire in TLR-Ligand Challenged Bubaline PBMCs as a Model of Bacterial and Viral Infection
Source: PLoS One. 2016 Jun 3;11(6):e0156598. doi: 10.1371/journal.pone.0156598 (PMC4892552; doi:10.1371/journal.pone.0156598)
Supplement: S4 Table — (DOCX) [file pone.0156598.s004.docx]

**S4 Table. List of the novel miRNAs that have hairpin sequences matched to the miRBase hairpin sequences**

| **SN** | **Arbitrary Id** | **Sequence (5’-3’)** | **Hairpin Sequence**  **(5’-3’)** | **Position** | **miRBase_Mature1_Acc** | **miRBase_ Mature1_ ID** | **miRBase_ Mature1_ Seq**  **(5’-3’)** |
| --- | --- | --- | --- | --- | --- | --- | --- |
| 1 | bta-miR-11033 | acuccauuuguuuugaugaug | gaggACUCCAUUUGUUUUGAUGAUGgauucuuacgcuccaucaucgucucaaaugagucuuc | 5 to 25 | MIMAT0013128 | eca-miR-136 | acuccauuuguuuugaugaugg |
| 2 | bta-miR-12051 | uagcuuaucagacugauguug | ugucgggUAGCUUAUCAGACUGAUGUUGacuguugaaucucauggcaacagcagucgaugggcugucugaca | 8 to 28 | MIMAT0003528 | bta-miR-21-5p | uagcuuaucagacugauguugacu |
| 3 | bta-miR-16059 | ccaauuuuccauguuccugugc | gauuggcauaggaacauggaagauugucagucaucaucuauuucugCCAAUUUUCCAUGUUCCUGUGCcaguc | 47 to 68 | MIMAT0012058 | bta-miR-2468 | auaggaacauggaagauuguca |
