# Supplementary material for: Identification of the MicroRNA Repertoire in TLR-Ligand Challenged Bubaline PBMCs as a Model of Bacterial and Viral Infection
Source: PLoS One. 2016 Jun 3;11(6):e0156598. doi: 10.1371/journal.pone.0156598 (PMC4892552; doi:10.1371/journal.pone.0156598)
Supplement: S5 Table — (DOCX) [file pone.0156598.s005.docx]

**S5 Table. List of the novel miRNAs (with respect to taurine miRNAs) expressed commonly in all the groups of treatment and control.**

| **Arbitrary Id** | **Sequence (5’-3’)** | **Hairpin Sequence (5’-3’)** | **Position** |
| --- | --- | --- | --- |
| bta-miR-11039 | ggcugguccgaugguagugggu | GGCUGGUCCGAUGGUAGUGGGUuaccagaacuuauuaacguuagugucacuaaaguugguauacaaccccccacugcuaaauuugacuggcu | 1 to 22 |
