# Supplementary material for: Identification of the MicroRNA Repertoire in TLR-Ligand Challenged Bubaline PBMCs as a Model of Bacterial and Viral Infection
Source: PLoS One. 2016 Jun 3;11(6):e0156598. doi: 10.1371/journal.pone.0156598 (PMC4892552; doi:10.1371/journal.pone.0156598)
Supplement: S6 Table — (DOCX) [file pone.0156598.s006.docx]

**S6 Table.** List of known and novel miRNAs of TLR ligand stimulated and non-stimulated control samples having fold change more than 2.

| **miRNAs** | **Fold change**  **(S-1 vs S-4)*** | **miRNAs** | **Fold change**  **(S-2 vs S-5)*** | **miRNAs** | **Fold change**  **(S-3 vs S-6)*** |
| --- | --- | --- | --- | --- | --- |
| ***Known miRNAs:*** | | | | | |
| bta-miR-421 | 19.5 Up | bta-miR-376e | 4.87 Up | bta-miR-106a | 2.49 Up |
| bta-miR-6529a | 15.17 Up | bta-miR-29d-5p | 1.94 Up | bta-miR-138 | 2.44 Up |
| bta-miR-194 | 12.39 Up | bta-miR-29c | 2.19 Down | bta-miR-99b | 2.01 Down |
| bta-miR-181a | 4.49 Up | bta-miR-181a | 2.29 Down | bta-let-7c | 2.07 Down |
| bta-miR-29c | 4.45 Up | bta-miR-421 | 3.04 Down | bta-miR-181a | 2.09 Down |
| bta-miR-30a-5p | 3.68 Up | bta-miR-671 | 4.96 Down | bta-miR-379 | 2.36 Down |
| bta-miR-320a | 3.55 Up | bta-let-7i | 10.81 Down | bta-miR-6529a | 2.45 Down |
| bta-let-7c | 3.22 Up | - | - | bta-miR-30a-5p | 4.57 Down |
| bta-miR-148b | 3.11 Up | - | - | bta-miR-34b | 5.05 Down |
| bta-miR-147 | 2.99 Up | - | - | bta-miR-29c | 13.36 Down |
| bta-miR-186 | 2.82 Up | - | - | bta-miR-671 | 28.43 Down |
| bta-miR-141 | 2.79 Up | - | - | bta-let-7i | 87 Down |
| bta-miR-138 | 2.78 Up | - | - | bta-miR-186 | 19.38 Down |
| bta-miR-132 | 2.77 Up | - | - | - | - |
| bta-miR-34b | 2.64 Up | - | - | - | - |
| bta-miR-769 | 2.55 Up | - | - | - | - |
| bta-miR-379 | 2.27 Up | - | - | - | - |
| bta-miR-15b | 2.01 Up | - | - | - | - |
| bta-miR-29d-5p | 3.01 Down | - | - | - | - |
| ***Novel miRNAs:*** | | | | | |
| bta-miR-11030 | 2.43 Up | bta-miR-12087 | 3.56 Up | bta-miR-11001 | 5.09 Up |
| bta-miR-11050 | 2.11 Up | bta-miR-11026 | 2.32 Up | bta-miR-11024 | 2.28 Down |
| bta-miR-11056 | 18.38 Up | bta-miR-11056 | 2.35 Down | bta-miR-13038 | 2.72 Down |
| bta-miR-11049 | 2.23Down | bta-miR-11036 | 3.07 Down | bta-miR-12013 | 4.17 Down |
| - | - | bta-miR-12005 | 7.64 Down | bta-miR-12096 | 47 Down |

*S1- LPS stimulated PBMC, 6hr; S4- Control, 6hr

S2- Poly IC stimulated PBMC, 12 hr; S5- Control, 12hr

S3- CpG stimulated PBMC, 12 hr; S6- Control, 12hr
